# Agreement between gastrointestinal panel testing and standard microbiology methods for detecting pathogens in suspected infectious gastroenteritis: Test evaluation and meta-analysis in the absence of a reference standard

**DOI:** 10.1371/journal.pone.0173196

**Published:** 2017-03-02

**Authors:** Karoline Freeman, Alexander Tsertsvadze, Sian Taylor-Phillips, Noel McCarthy, Hema Mistry, Rohini Manuel, James Mason

**Affiliations:** 1 Warwick Medical School, University of Warwick, Coventry, United Kingdom; 2 NIHR Health Protection Research Unit in Gastrointestinal Infections, Oxford, United Kingdom; 3 NIHR Health Protection Research Unit in Gastrointestinal Infections, London, United Kingdom; Universita degli Studi di Parma, ITALY

## Abstract

**Objective:**

Multiplex gastrointestinal pathogen panel (GPP) tests simultaneously identify bacterial, viral and parasitic pathogens from the stool samples of patients with suspected infectious gastroenteritis presenting in hospital or the community. We undertook a systematic review to compare the accuracy of GPP tests with standard microbiology techniques.

**Review methods:**

Searches in Medline, Embase, Web of Science and the Cochrane library were undertaken from inception to January 2016. Eligible studies compared GPP tests with standard microbiology techniques in patients with suspected gastroenteritis. Quality assessment of included studies used tailored QUADAS-2. In the absence of a reference standard we analysed test performance taking GPP tests and standard microbiology techniques in turn as the benchmark test, using random effects meta-analysis of proportions.

**Results:**

No study provided an adequate reference standard with which to compare the test accuracy of GPP and conventional tests. Ten studies informed a meta-analysis of positive and negative agreement. Positive agreement across all pathogens was 0.93 (95% CI 0.90 to 0.96) when conventional methods were the benchmark and 0.68 (95% CI: 0.58 to 0.77) when GPP provided the benchmark. Negative agreement was high in both instances due to the high proportion of negative cases. GPP testing produced a greater number of pathogen-positive findings than conventional testing. It is unclear whether these additional ‘positives’ are clinically important.

**Conclusions:**

GPP testing has the potential to simplify testing and accelerate reporting when compared to conventional microbiology methods. However the impact of GPP testing upon the management, treatment and outcome of patients is poorly understood and further studies are needed to evaluate the health economic impact of GPP testing compared with standard methods.

The review protocol is registered with PROSPERO as CRD42016033320.

## Introduction

Gastroenteritis is a common, transient, mostly self-limiting disorder usually caused by infection with viruses, bacteria or parasites. Identifying the infectious agent in severe cases may aid decision making in terms of treatment, isolation, management, and further investigations. Standard laboratory methods include culture for bacteria, nucleic acid amplification and immunoassays for viruses and microscopy or enzyme immunoassays for parasites as well as culture for amoeba. Tests have turnaround times of up to three days and in practice recommendations for routine screening of stool samples for people with diarrhoea, vomiting and abdominal pain are for a limited range of pathogens in line with the Public Health England syndromic algorithm [1] although the number of pathogens actually tested for varies. The algorithm prescribes testing in two stages aiming to rule out common gastrointestinal pathogens. The number and type of pathogens tested for depends on the setting (hospital versus community), season, as well as whether patients are children or travellers [1]. Gastrointestinal pathogen panel (GPP) tests offer a more extensive range of pathogens than is covered by the algorithm with some variation between panels. GPP tests exploit multiplex nucleic acid amplification methodology, testing for a wide range of bacteria, viruses and parasites in a single run, potentially increasing the throughput and volume of information from one test run and decreasing reporting times to a day or less. Systems differ considerably in the number of samples that can be run simultaneously.

Adequate evaluation of GPP tests is important as the tests gradually diffuse into routine clinical practice. Normally, a reference method is selected that identifies the true infectious cause and provides a standard with which to assess alternative tests, assessing their test sensitivity and specificity. Ideally such a reference standard is incontrovertibly accurate and independent. However neither GPP nor conventional testing can be assumed to have greater accuracy in identifying clinically important pathology, and no further test has been identified to act as an independent reference standard. Polymerase chain reaction (PCR) can detect pathogen DNA at very low levels including from non-viable organisms, generally leading to more test positive outcomes, but of uncertain clinical importance. In the absence of a reference standard, or an adequate resolving test for discrepant analysis, sensitivity and specificity cannot be calculated [2].

In such circumstances the Food and Drug Administration (FDA) recommends reporting measures of positive and negative test agreement without further validation of discordant or concordant test results in the Statistical Guidance on Reporting Results from Studies Evaluating Diagnostic Tests [3] while the Quality Assessment of Diagnostic Accuracy Studies (QUADAS 2) tool [4] classifies primary studies of test accuracy as being at high risk of bias when an inadequate reference standard is used. While currently available evidence on test accuracy limits the interpretation of results and usefulness to decision makers, exploring test agreement by taking each of the tests in turn as the benchmark test (rather than reference standard) can highlight differences between the tests. A systematic review of the clinical effectiveness of GPP testing when compared to standard microbiology laboratory methods was undertaken in support of decision making about the adoption of GPP testing in patients with symptoms suggestive of infectious gastroenteritis presenting at a community or hospital setting.

## Methods

This review forms part of a broader Health Technology Assessment (HTA) report by the same authors.

### Search strategy

Multiple electronic database searches were undertaken by a qualified information specialist from inception to January 2016 including searches in Medline, Embase, Web of Science and Cochrane Database of Systematic Reviews with supplementary searches of other online resources. The search combined subject headings and free text words including terms for gastroenteritis and multiplex polymerase chain reaction. Full searches are available in S1 File. Reference lists of all reviews and included studies were screened and trial websites were searched for ongoing studies. Authors were contacted to seek clarification on study populations when necessary.

### Study eligibility criteria

Studies of adults and children with suspected gastroenteritis comparing GPP tests with comprehensive coverage of bacteria, viruses and parasites with standard microbiology techniques reporting test performance, patient management, clinical and patient reported outcomes were included. The setting considered was clinical laboratories receiving samples from primary and secondary care. Eligible study designs followed a hierarchy of best available evidence with the most desirable being 1) test-treat trials comparing clinically relevant outcomes (e.g., morbidity, mortality, length of stay and length of isolation) for patients randomised to either conventional testing or GPP. This was followed by 2) clinical diagnostic test accuracy studies that compare the index tests (GPP) and the comparator (standard microbiology methods) to an adequate reference standard, 3) studies that compare discrepant results between the index tests (GPP) and the comparator (standard microbiology methods) using an unbiased umpire test [2], 4) studies of agreement and disagreement between the index tests (GPP) and the comparator without using an unbiased umpire test and 5) studies of head to head comparisons of different index tests (GPP) reporting agreement of tests. Only studies that reported sufficient raw data to calculate positive and negative agreement by pathogen were considered.

Studies were excluded if they considered partial tests with coverage of less than the three groups of pathogens and if no positive and negative agreement by pathogen could be determined. Additionally, reviews, biological studies, case reports, editorials and opinions, poster presentations without supporting abstracts, non-English language reports, and meeting abstracts without sufficient numerical detail on test performance per pathogen were excluded.

### Study selection

Two reviewers independently screened the titles and abstracts of all records identified by the searches. Full texts of all studies deemed potentially relevant were obtained and two reviewers independently assessed these for inclusion. Discrepancies at both stages were resolved through discussion.

### Data extraction

Test results for GPP and standard microbiology methods were extracted at the pathogen level into two-by-two contingency tables following the format in Table 1.

**Table 1:** Contingency table of test agreement.

	Comparator +	Comparator -
GPP +	a) +/+	b) -/+
GPP -	c) +/-	d) -/-

### Assessment of risk of bias and applicability

Quality assessment of included studies was undertaken by two independent reviewers and used tailored QUADAS-2 [4]. Quality assessment assessed the risk of bias and applicability concerns for included studies at the pathogen level where the GPP method was the index test, conventional methods were the comparator and any efforts to verify discordant results were assessed under the reference standard domain. The main adaptation of the QUADAS-2 tool consisted of the addition of a domain for the comparator. The eligible studies compared GPP testing to a comparator which consisted broadly speaking of a range of standard microbiology tests which cannot be classed as the reference standard because GPP testing may be superior to standard microbiology methods. Therefore, the comparator was assessed in addition to the index test and the reference standard. Similar signalling questions in terms of blinding and threshold as for the index test were considered for the comparator. Furthermore, we added signalling questions to the ‘reference standard’ as well as the ‘flow and timing’ domain requiring for a low risk of bias judgment, that the verification methods used in the studies were independent and unbiased, all discordant results rather than a proportion had received verification and that all samples had received the comparator methods for all pathogens considered in the study.

### Data synthesis

In the absence of a reference standard we calculated positive agreement (a/a+c) and negative agreement (d/b+d) when benchmarked against the comparator (mainly standard microbiology methods) and positive agreement (a/a+b) and negative agreement (d/c+d) when benchmarked against GPP for each pathogen using methods outlined in the Statistical Guidance on Reporting Results from Studies Evaluating Diagnostic Tests by the FDA [3]. This is equivalent to measuring the sensitivity and specificity of GPP using standard microbiology methods as the reference standard, and the sensitivity and specificity of standard microbiology methods using GPP as the reference standard. Positive and negative agreements, using the two benchmarks, were then meta-analysed by pathogen if the denominator was ≥20 using random effects meta-analysis of proportions using the metaprop command in Stata SE 14.1 [5] and reported in tables and Forest plots. Methods for bivariate analysis of diagnostic tests findings were not used because of restrictive requirements for the number of studies within each pathogen (minimum of 4 studies with complete data). Exact binomial methods were used to estimate 95% confidence intervals using the Freeman-Tukey transformation of proportions, and the I^2^ statistic of between study heterogeneity was computed. Data verifying discordant results was tabulated to explore the option of discrepant analysis using a suitable resolving test [6].

## Results

**Fig 1:**
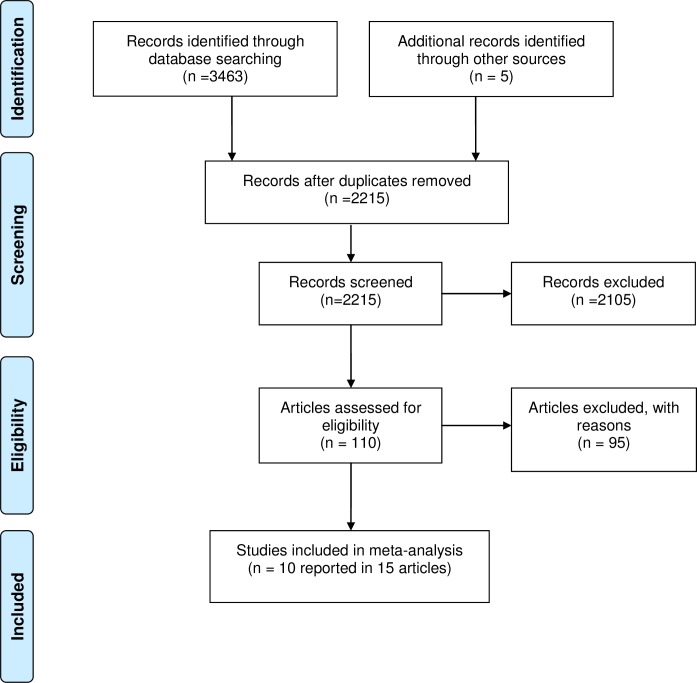
PRISMA flow diagram of study selection.

The search identified 3468 records. Following duplicate removal, we screened 2215 unique records of which 110 were taken forward to full text assessment. Ten studies contributed sufficient data to calculate positive and negative agreement and be included in the meta-analysis [7–16]. The PRISMA diagram of study selection is provided in Fig 1.

### Study characteristics

Included studies only represented study designs 4 and 5 described in the methods section. Studies were heterogeneous in terms of participants included (hospital versus community, risk, comorbidities), country of origin (developing versus developed), standard microbiology methods used and number and type of pathogens considered (see S1 Table for study characteristics). Of the 10 studies eight evaluated the xTAG GPP test, one study evaluated the FilmArray GPP test and a further study evaluated both tests.

### Risk of bias and concerns regarding applicability of study findings

The risk of bias of the included studies of test accuracy was generally high (S2 Table). None of the studies used a reference standard against which the GPP tests and standard microbiology methods could be reliably evaluated. Instead, in most studies, the GPP tests were compared against the standard microbiology methods, biasing the assessment. Discrepant results between GPP test and standard microbiology methods were verified at the pathogen level in 4/10 studies, although confirmatory tests were not adequately independent of GPP and/or standard microbiology tests. In many cases, the standard microbiology methods were not performed for all pathogens covered by the GPP test. There were concerns about the applicability and relevance of standard microbiology methods and verification tests used in the majority of studies, in reference to routine clinical practice.

### Test performance

Pooled estimates of positive and negative agreement by pathogen and overall for all pathogens between GPP and standard microbiology methods are given in Table 2 (standard microbiology methods provide the benchmark) and Table 3 (GPP test provides the benchmark). Contingency tables of the raw data of test agreement by pathogen informing the meta-analysis are provided in S3 Table. Overall, more studies contributed to the calculation of negative than positive agreement as only studies with sufficient numbers (denominator ≥20) were included in the analysis and the presence of pathogens was a rare event. For a number of pathogens, *E*. *coli* O157, ETEC,STEC, *Vibrio cholera*, *Yersinia enterocolitica* and *Entamoeba histolytica* (rare pathogens or no test requested by physician and marked as empty rows in the Tables 2 and 3) limited data were available and no positive agreement could be estimated.

**Table 2:** Positive and negative agreement: xTAG vs. standard microbiology methods (benchmark).

**Positive Agreement:**	**RE**	**LCI**	**UCI**	**N**	**Q**	**p**	**I^2^**
*C*. *difficile*	0.959	0.933	0.980	5	5.9	0.207	32%
*Campylobacter*	0.959	0.924	0.985	6	8.0	0.157	37%
*E*. *coli* O157	-	-	-	-	-	-	-
ETEC	-	-	-	-	-	-	-
STEC	-	-	-	-	-	-	-
*Salmonella*	0.818	0.666	0.934	5	30.8	0.000	87%
*Shigella*	0.989	0.949	1.000	3	3.6	0.164	45%
*Vibrio cholerae*	-	-	-	-	-	-	-
*Yersinia enterocolitica*	-	-	-	-	-	-	-
Adenovirus	0.558	0.413	0.699	-	-	-	-
Norovirus	0.927	0.893	0.956	7	10.9	0.093	45%
Rotavirus	0.958	0.920	0.985	3	2.9	0.240	30%
*Cryptosporidium*	0.914	0.794	0.989	1	-	-	-
*Entamoeba histolytica*	-	-	-	-	-	-	-
*Giardia*	1.000	0.935	1.000	1	-	-	-
**Negative Agreement:**	**RE**	**LCI**	**UCI**	**N**	**Q**	**p**	**I**^**2**^
*C*. *difficile*	0.968	0.933	0.991	7	128.0	0.000	95%
*Campylobacter*	0.968	0.950	0.982	10	83.7	0.000	89%
*E*. *coli* O157	0.995	0.990	0.998	6	10.8	0.055	54%
ETEC	0.988	0.964	1.000	4	23.7	0.000	87%
STEC	0.990	0.984	0.995	4	2.8	0.418	0%
*Salmonella*	0.940	0.866	0.986	10	726.0	0.000	99%
*Shigella*	0.985	0.965	0.997	8	120.0	0.000	94%
*Vibrio cholerae*	1.000	0.998	1.000	4	0.1	0.988	0%
*Yersinia enterocolitica*	1.000	1.000	1.000	4	0.4	0.933	0%
Adenovirus	0.990	0.983	0.996	2			-
Norovirus	0.969	0.944	0.987	12	239.0	0.000	95%
Rotavirus	0.991	0.979	0.999	8	36.7	0.000	81%
*Cryptosporidium*	0.989	0.954	1.000	5	77.4	0.000	95%
*Entamoeba histolytica*	0.991	0.979	0.998	5	20.5	0.000	81%
*Giardia*	0.989	0.970	0.999	7	46.4	0.000	87%
**Overall Agreement:**	**RE**	**LCI**	**UCI**	**N**	**Q**	**p**	**I**^**2**^
Positive	0.929	0.898	0.955	33	188.3	0.000	83%
Negative	0.982	0.976	0.988	101	2080.8	0.000	95%

RE: Random effect estimate, measure of agreement; LCI: lower confidence interval; UCI: upper confidence interval; N: number of studies contributing; Q, p, I^2^: Heterogeneity Cochrane Q statistic, p-value and I^2^ index

**Table 3:** Positive and negative agreement: Standard microbiology methods vs. xTAG (Benchmark).

**Positive Agreement:**	**RE**	**LCI**	**UCI**	**N**	**Q**	**p**	**I**^**2**^
*C*. *difficile*	0.801	0.594	0.948	5	124.0	0.000	97%
*Campylobacter*	0.639	0.398	0.849	7	167.0	0.000	96%
*E*. *coli* O157	0.750	0.534	0.920	1	-	-	-
ETEC	-	-	-	-	-	-	-
STEC	-	-	-	-	-	-	-
*Salmonella*	0.484	0.278	0.693	8	173.0	0.000	96%
*Shigella*	0.734	0.381	0.971	3	61.6	0.000	97%
*Vibrio cholerae*	-	-	-	-	-	-	-
*Yersinia enterocolitica*	-	-	-	-	-	-	-
Adenovirus	0.570	0.425	0.710	2	-	-	-
Norovirus	0.774	0.584	0.920	8	215.0	0.000	97%
Rotavirus	0.924	0.853	0.975	3	6.5	0.039	69%
*Cryptosporidium*	0.508	0.407	0.608	2	-	-	-
*Entamoeba histolytica*	-	-	-	-	-	-	-
*Giardia*	0.337	0.237	0.444	2	-	-	-
**Negative Agreement:**	**RE**	**LCI**	**UCI**	**N**	**Q**	**p**	**I**^**2**^
*C*. *difficile*	0.996	0.992	0.999	7	11.1	0.084	46%
*Campylobacter*	0.998	0.994	1.000	10	37.4	0.000	76%
*E*. *coli* O157	1.000	1.000	1.000	6	3.4	0.637	0%
ETEC	0.999	0.996	1.000	4	3.0	0.393	0%
STEC	1.000	0.998	1.000	4	4.9	0.182	38%
*Salmonella*	0.992	0.980	0.999	10	94.4	0.000	91%
*Shigella*	1.000	0.999	1.000	8	12.0	0.099	42%
*Vibrio cholerae*	1.000	0.999	1.000	4	3.5	0.326	13%
*Yersinia enterocolitica*	1.000	0.999	1.000	4	0.4	0.933	0%
Astrovirus	0.989	0.971	0.999	7	68.7	0.000	91%
Norovirus	0.995	0.990	0.998	12	34.9	0.000	69%
Rotavirus	0.998	0.992	1.000	8	29.3	0.000	76%
*Cryptosporidium*	1.000	0.999	1.000	5	2.0	0.743	0%
*Entamoeba histolytica*	1.000	0.999	1.000	5	3.5	0.481	0%
*Giardia*	1.000	1.000	1.000	7	1.8	0.941	0%
**Overall Agreement**	**RE**	**LCI**	**UCI**	**N**	**Q**	**p**	**I**^**2**^
Positive	0.678	0.580	0.770	41	1340.5	0.000	97%
Negative	0.998	0.997	0.999	101	429.2	0.000	77%

RE: Random effect estimate, measure of agreement; LCI: lower confidence interval; UCI: upper confidence interval; N: number of studies contributing; Q, p, I^2^: Heterogeneity Cochrane Q statistic, p-value and I^2^ index

#### Standard microbiology methods providing the benchmark

Meta-analysis showed that when standard microbiology methods provided the benchmark, virtually all positive cases found by xTAG were confirmed by conventional testing leading to high levels of positive agreement findings (0.93 [95% CI 0.90 to 0.96]) (Table 2). Additional positives identified by xTAG were few compared to the vast majority of specimens that are pathogen-negative, thus negative agreement remained high (0.98 [95% CI 0.98 to 0.99]). Although overall findings were indicative, they nonetheless feature an equal weighting of pathogen-level findings not reflecting the prevalence of individual pathogens.

**Fig 2:**
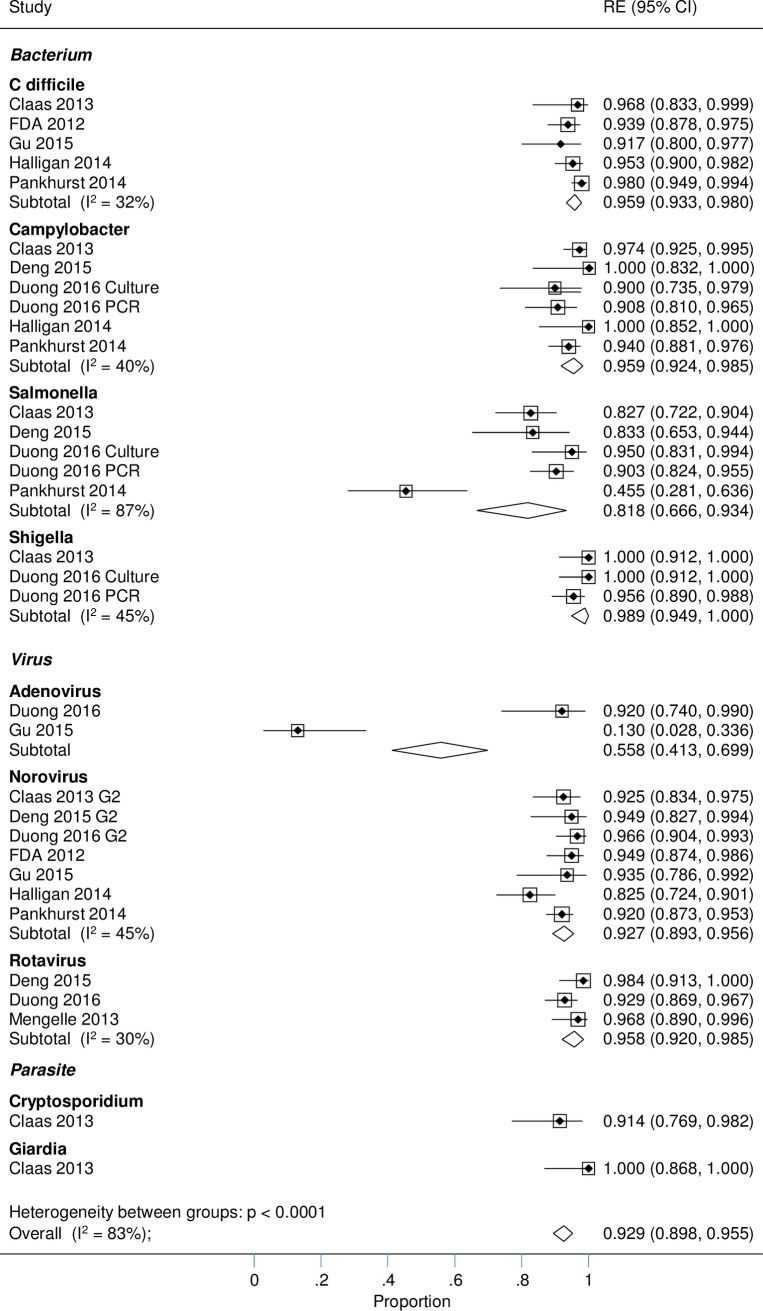
Positive agreement: xTAG vs. conventional testing (benchmark).

There was generally little variation between pathogens for both positive and negative agreement. Positive agreement for adenovirus was an exception where positive agreement was considerably lower at 0.56. This is visualised in the Forest plot in Fig 2. Gu et al. 2015 [13] reported that an additional 20 samples positive for adenovirus detected by comparator were due to the use of multiplex PCR, which detected all serotypes while xTAG only detected adenovirus 40/41 resulting in the poor agreement of tests for this virus. The outlying finding for *Salmonella* (Pankhurst et al., 2014 [16]), caused by a high number of missed *Salmonella* infections by xTAG, could not be explained.

Generally, both tests agreed about the absence of pathogens, masking the relatively small number of disagreements. The Forest plot shows, however, that there were a few outliers where studies report a significantly higher number of positives for certain pathogens with xTAG compared to standard microbiology methods, specifically *Campylobacter* and norovirus in a small study of 49 adult kidney transplant recipients [9] and *Salmonella* in a study where bacteria were tested by PCR as well as culture.[11] (Figure A in S1 Figs)

Used as a measure of overall heterogeneity of estimates, I^2^ is moderate (for positive agreement) to high (for negative agreement) at the pathogen level.

In summary, using standard microbiology methods as benchmark the impression was that GPP testing and standard microbiology methods provide very similar results.

#### GPP testing providing the benchmark

**Fig 3:**
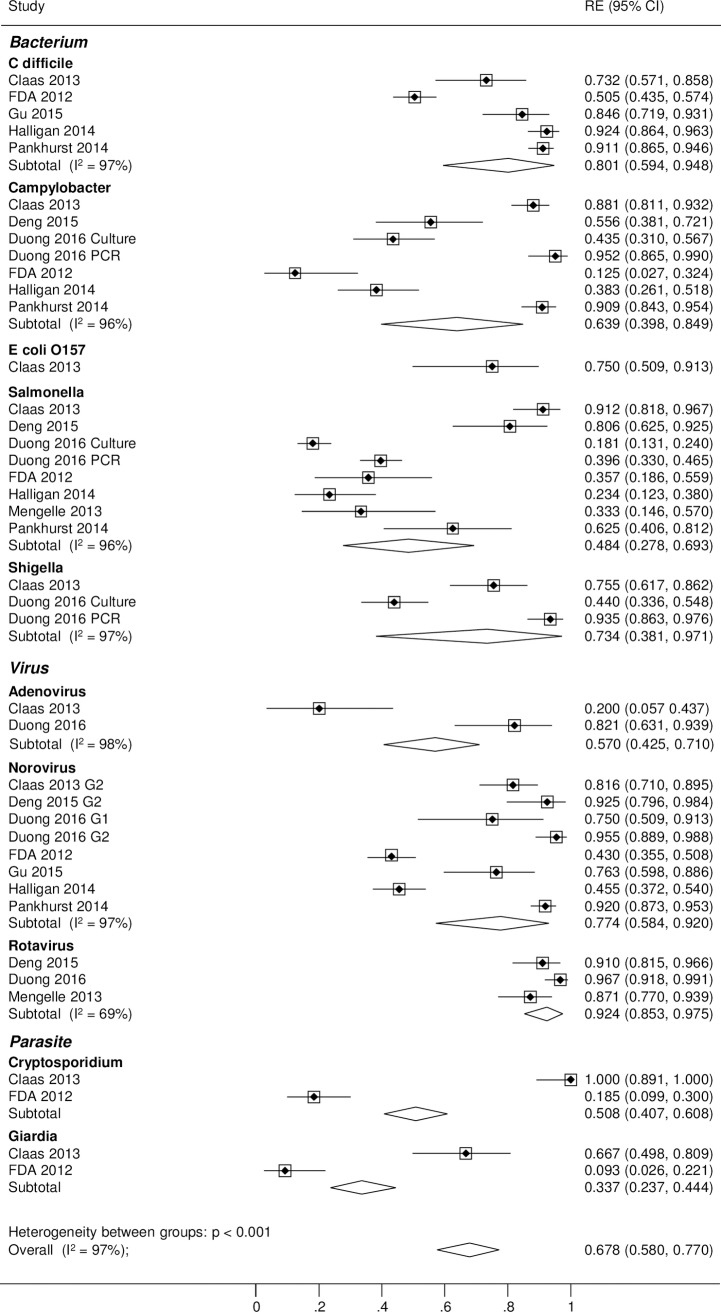
Positive agreement: Conventional testing vs. xTAG (Benchmark).

Levels of agreement when xTAG provided the benchmark are shown in Table 3.The positive agreement between xTAG and conventional methods was considerably reduced when xTAG provided the benchmark. The Forest plot in Fig 3 visualises the inconsistency and variation in positive agreement between studies across all pathogens. When xTAG was the benchmark, the positive cases ‘missed’ by standard microbiology methods had a considerable impact on the positive agreement findings.

Since the overall positive agreement is 0.68 (95% CI: 0.58 to 0.77), inverting these figures means that xTAG finds about 1.5 times more positive results (95% CI: 1.3 to 1.7). Negative agreement was consistently very high across studies and pathogens (Figure B in S1 Figs) with the exception of the Gu et al. (2015) study [13] discussed previously. Heterogeneity was moderate to high when considering I^2^ and was higher for positive agreement than for negative agreement. Using the GPP test as the benchmark, it becomes clear GPP testing detects significantly more pathogens.

Only two studies [7 13] contributed data to the meta-analytic evaluation of the FilmArray GPP test with detailed outcomes reported in a recent HTA report by the same authors. Qualitatively these findings were similar to the findings reported here for the xTAG GPP test.

### Verification of discordant results

In the absence of a reference standard to verify test results, four studies [7 10 12 16] verified discordant pathogen findings by pathogen that did not agree when tested by GPP and conventional methods (S4 Table). Verification methods were PCR based. Even though verification methods differed from standard microbiology methods and used different molecular targets when compared to GPP assays, they could not be considered independent. GPP assays essentially use PCR and other PCR-based methods would be expected to resolve discordant results in their favour. A further complexity was that sometimes conventional methods included some use of PCR. Discordant analysis of GPP positive/ standard microbiology methods negative generally favoured GPP as anticipated, however analysis of GPP negative/ standard microbiology method outcomes more often favoured standard microbiology methods (S4 Table). Discordant analysis failed to resolve all discordant samples; for a considerable number of discordant results, discrepant analysis did not help to identify the underlying cause of the discrepancy. No particular pattern for any specific pathogen emerged from the discordant analyses.

## Discussion

### Principal study findings

Our meta-analysis of ten primary studies comparing GPP testing with standard microbiology methods reports the range of possible outcomes of positive agreement when each method in turn was considered the benchmark. Positive agreement ranged from 0.93 (95% CI 0.90 to 0.96) when conventional methods provided the benchmark to 0.68 (95% CI: 0.58 to 0.77) when GPP provided the benchmark while negative agreement was consistently high. This was due to the large number of negative results agreed upon by both methods. No previous systematic review of GPP tests was identified in our review, but assuming the use of conventional testing as a reference standard would mask issues with the use of GPP tests. In particular, gastrointestinal pathogen panel tests generate significantly more additional positive results. These, however, are of uncertain clinical importance in the absence of an appropriate reference standard or a suitable resolver test for discrepant analysis. On the other hand high positive agreement when conventional methods provided the benchmark could not be consistently shown by all studies for all pathogens suggesting that GPP performance differs for different pathogens. Pankhurst et al. (2014) [16] reported a great number of additional positives with conventional laboratory methods for *Salmonella spp*. which were missed with molecular based tests. Poor detection of some pathogens in some studies requires further investigation and assessment of which assays should be reported within a GPP.

### Strengths and weaknesses

In the absence of more robust methods of assessing test accuracy, positive and negative agreements have been produced against a benchmark, as recommended by FDA guidance. [3] The meta-analytic outcomes reported here are of exploratory nature summarising the available evidence, illustrating patterns in the data and describing heterogeneity. Test agreement is not a measure of test accuracy as it neither considers either approach as the ‘truth’ nor does it consider if both tests while agreeing are actually wrong. Rather, by varying the benchmark, different views about the level of agreement between tests are explored. Findings were typically heterogeneous, probably reflecting in part methodological and statistical heterogeneity and drawing from studies of variable quality. High levels of heterogeneity for negative agreement were partly driven by variations in large and very precise study estimates. Additionally, a high I^2^ was possibly caused by differences in thresholds used across studies. We report I^2^ as a statistical measure of between study heterogeneity which is not often used in test accuracy studies because it does not account for heterogeneity due to a threshold effect. As the studies included a number of different standard microbiology tests and did not report thresholds it is difficult to judge to what extent a threshold effect existed. Therefore the finding of high levels of heterogeneity is of interest and we have listed a number of reasons that could explain this heterogeneity but cannot exclude that differences in threshold caused at least some of this heterogeneity.

The presence of very small sample sizes and heterogeneity creates particular methodological problems for meta-analyses of diagnostic test performance. A textbook approach might include all studies (large and small) regardless of patient numbers and assess test accuracy using bivariate methods. However in the present analysis, inclusion of very small studies (<20) has a dramatic impact upon sparsely informed random effect models. Bivariate analyses are balanced, including studies contributing to sensitivity and specificity estimates of test accuracy. Additionally available routines require a minimum of four pathogen studies to work. Use of the bivariate approach would exclude a large number of informative negative agreement studies from the analyses presented in this paper. Pragmatically, the greater inclusivity of univariate analysis of agreement values was preferred to the theoretical correctness of bivariate analysis.

Analyses are presented at the pathogen level requiring independence assumptions both within and between pathogens, i.e. repeat samples of the same patient are not included and having one pathogen does not affect the likelihood of having another pathogen. Pooled summary estimates (across pathogens) have been included for information. However, these pooled estimates are not weighted by the prevalence of the different pathogens, and include varying multiple usage of samples where studies have tested samples with varying components of the conventional panel of tests, thus violating the independence assumption. Accepting these limitations and issues the summary findings remain qualitatively informative.

Within the clinical studies identified, many pathogens were present only at low prevalence, and the context of studies included a mixture of different patient populations (e.g. children, immunocompromised patients, community) each with their own distribution of prevalence of pathogens. This was not considered in sensitivity analyses due to the number of potential covariates (not always well quantified) and issues of multiplicity.

This review has evaluated GPP systems according to their current specification, but it is anticipated the coverage of these systems will continue to evolve in response to changing pathogen prevalence, hence the evaluation problem is a dynamic one.

### Meaning of a positive test outcome: Implications for clinicians and policymakers

If conventional methods accurately identify clinically important disease then GPP testing would correctly identify the same positive cases but add further false positive patients who may receive unnecessary treatment and potentially a delayed return to normal activities. However, if GPP testing is accurate (all of its positives are clinically important) then current testing misses clinically important pathogens, potentially resulting in under-treatment and impaired infection control measures. Expertise is important when identifying parasites by standard microbiology methods, where detection is dependent upon the life-cycle of the parasite and its appearance in the inhomogeneous stool sample. These difficulties may be overcome by using GPP testing. The consequence for clinical care is complicated since most infections are self-limiting and require no pathogen specific treatment, just hygiene, hydration and watchful waiting. Only for a select few pathogens is specific treatment recommended (*C*. *difficile*, some strains of *Salmonella*, *Shigella*, *E*. *coli* (non-STEC), *Campylobacter* and *Giardia*) although not all patients are treated. By reducing reporting from three to one days, GPP testing has the potential to streamline the management of non-infectious cases and use of hospital isolation rooms, intended to reduce the spread of infection. Currently there is no robust evidence to support changes in hospital care due to GPP testing and their plausibility is uncertain. For example, length of stay in hospital and use of isolation rooms may be primarily driven by comorbidity and hygiene rather than identification of infectious agents.

Currently the clinical importance of the additional pathogens identified by GPP testing is uncertain and there is concern that at least some of the additional findings are non-viable pathogens. GPP tests target microbial DNA and RNA. This will result in challenges associated with the interpretation of GPP test results in clinical practice, a concern shared by other authors [17]. Firstly, GPP tests cannot distinguish between viable and non-viable pathogens. The detection of microbial nucleic acid does not necessarily imply the presence of viable, replicating organisms responsible for disease. Secondly, many pathogens can exist asymptomatically (e.g., norovirus and *Salmonella* spp) or sub-clinically (e.g., *C*. *difficile* nontoxigenic strains) in a colonization-like status [18–20] where association with disease is unlikely. It is believed that this in part explains the increased number of positive results as well as the increased findings of co-infections with GPP testing. Further understanding is needed as to how these results should be interpreted. However the inclusiveness of panel tests may provide a gain for users, e.g. Enteroaggregative *E*. *coli* (EAEC) is not detected by routine culture.

In addition to providing pathogen-level analysis, studies typically reported overall levels of detection of pathogens with a GPP test compared to a battery of standard microbiology methods. Reporting the total number of pathogens detected may mislead by confounding greater GPP ‘sensitivity’ to detect specific pathogens with different coverage of pathogens by GPP and conventional methods. Concordance of methods depends on including a common number and type of pathogens tested. Thus discrepancies between GPP tests and conventional methods may result not only from differences in accuracy but also from differences in their respective targets. For example, discrepancies reported by Gu et al. (2015) [13] for adenovirus may have been due to comparator PCR identifying all adenovirus strains while the GPP system only identified adenovirus 40 and 41. Similarly Pankhurst et al. (2014) [16] reported that xTAG has two targets for C. difficile (genes for toxins A and B) and includes two primers against norovirus GI and GII strains. However, the single quantitative PCRs used as comparators in the study targeted only the gene for toxin B or the GII strain. This misalignment problem may be exacerbated when comparing different GPP systems with their varying coverage.

An additional limitation of GPP tests is that although the presence of bacterial pathogens is identified there is no bacterial culture to support either antimicrobial susceptibility testing or subtyping to support public health surveillance. Culturing from positive samples may therefore be required to guide antimicrobial treatment or public health investigation when these are required.

### Future research needs

Agreement measures are not measures of test performance in a conventional sense, and only adequately designed research will resolve uncertainties about the introduction of GPP testing. It may not be possible to design a study with an adequate independent reference standard. Molecular methods may not be the best option to address the problem, as the presence of pathogen DNA may not answer the question of the clinical importance of the identified pathogens. On the other hand there is widespread belief that conventional microbiology laboratory methodologies are going to be outperformed by new PCR based technology. A randomised controlled trial, randomising patients to conventional or GPP testing, would establish the relative clinical and cost-effectiveness outcomes of the different approaches.

## Supporting information

S1 Figs Meta-analytic outcomes of test agreement. (PDF)

S1 File Search strategy for clinical effectiveness review. (PDF)

S1 Table Overview of study characteristics of included studies. (PDF)

S2 Table Judgement of risk of bias and applicability of included studies. (PDF)

S3 Table 2x2 data by pathogen and GPP test. (PDF)

S4 Table Outcomes of verification of discordant results in 4 studies reporting outcomes of verification by pathogen using a third method. (PDF)
