# Supplementary material for: Agreement between gastrointestinal panel testing and standard microbiology methods for detecting pathogens in suspected infectious gastroenteritis: Test evaluation and meta-analysis in the absence of a reference standard
Source: PLoS One. 2017 Mar 2;12(3):e0173196. doi: 10.1371/journal.pone.0173196 (PMC5333893; doi:10.1371/journal.pone.0173196)

S1 Figs. Meta-analytic outcomes of test agreement

Figure A - Negative agreement: xTAG vs. standard microbiology methods (benchmark)

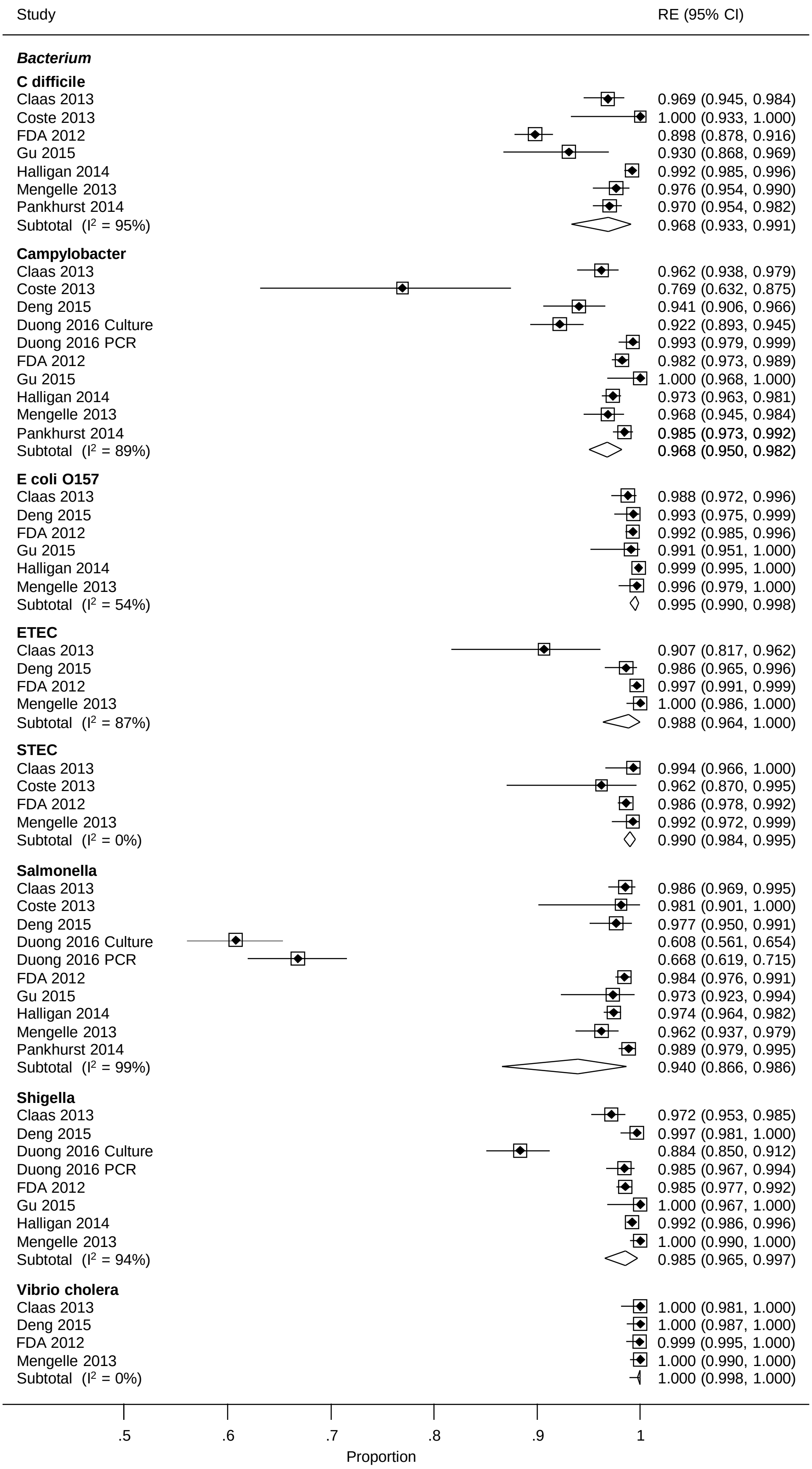

Negative agreement: xTAG vs. standard microbiology methods (benchmark) *(continued)*

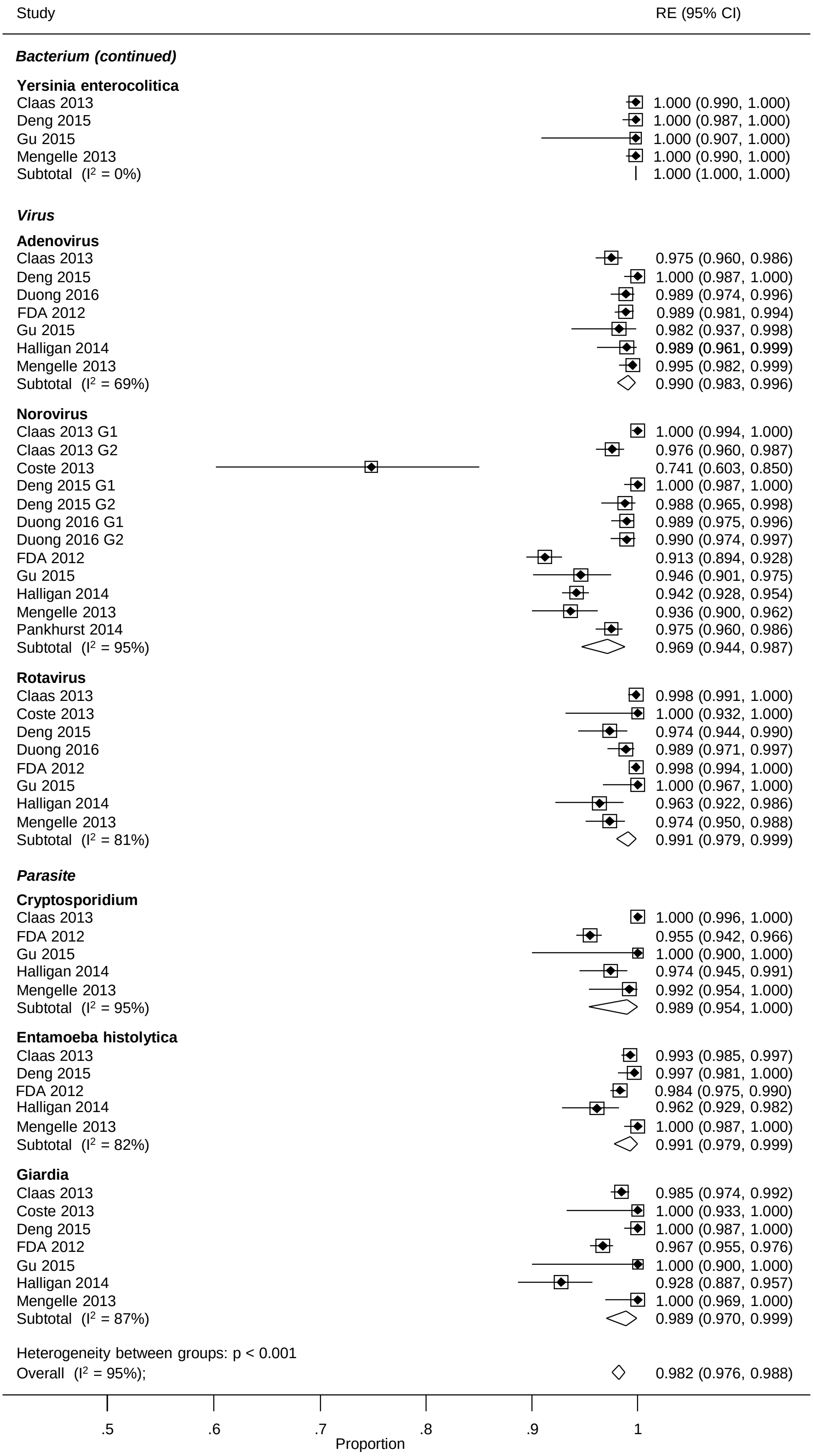

Figure B - Negative agreement: Standard microbiology methods vs. xTAG (Benchmark)

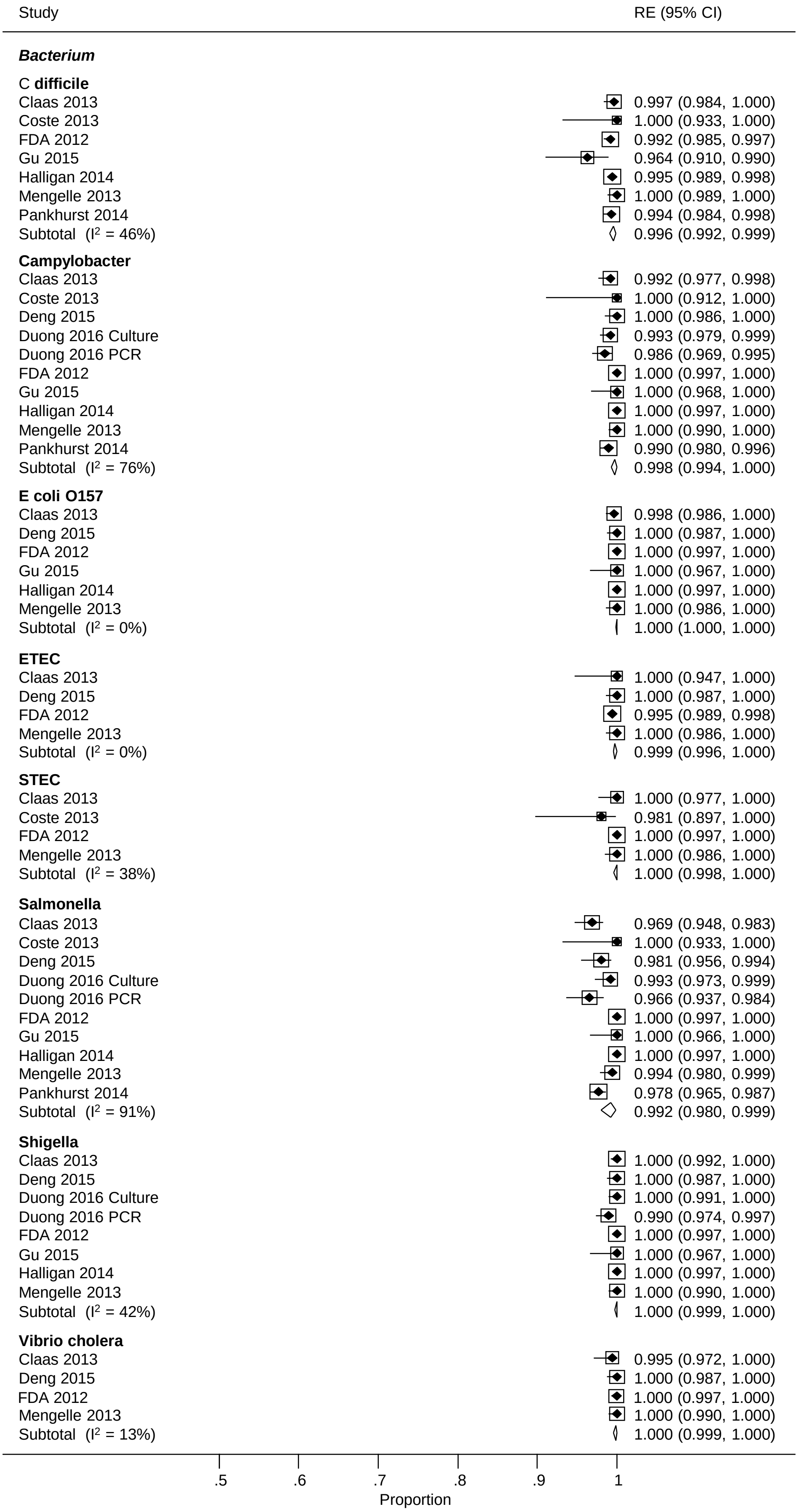

Negative agreement: Standard microbiology methods vs. xTAG (Benchmark) *continued*

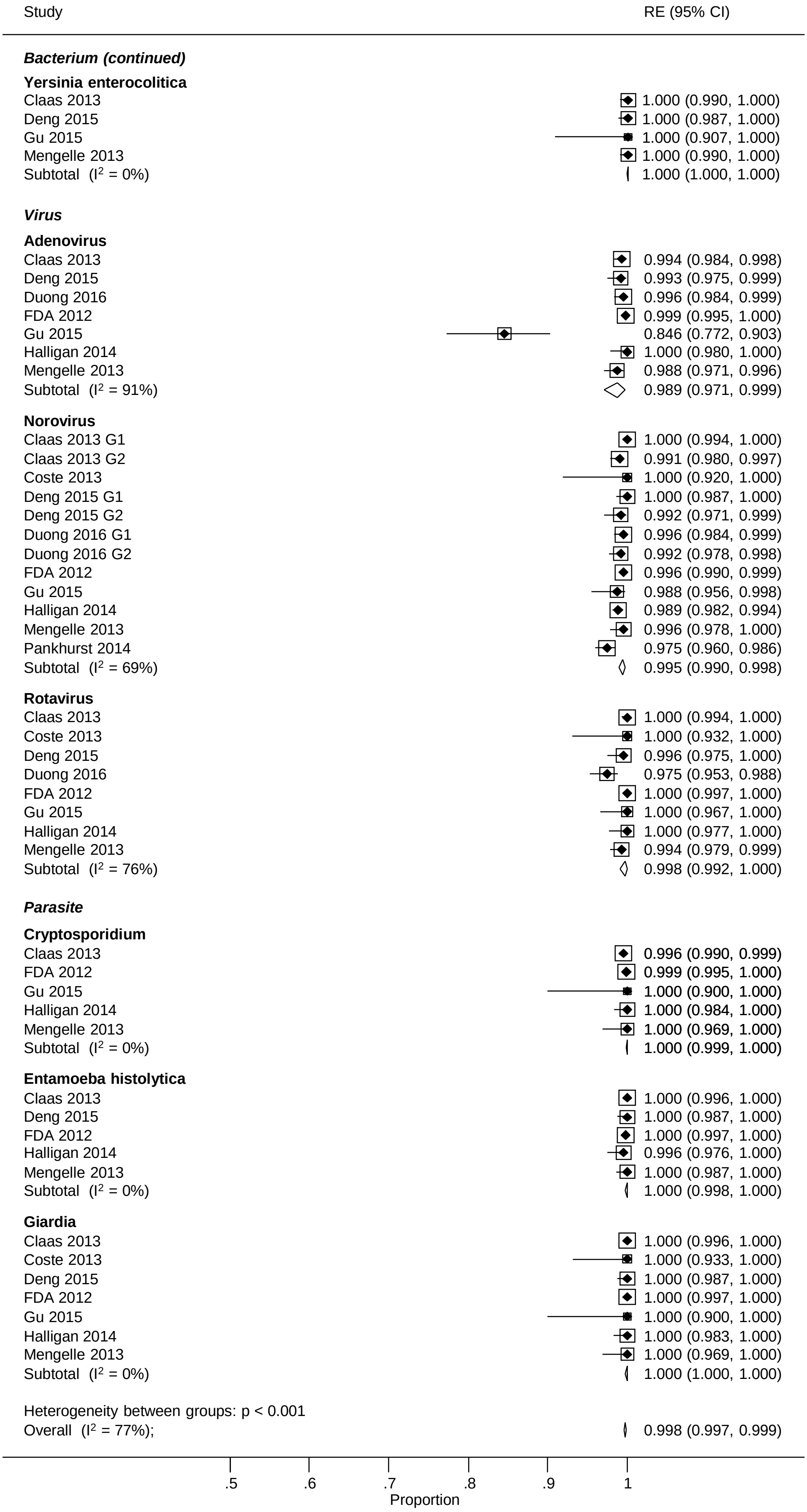

Supplement: S1 Figs — (PDF) [file pone.0173196.s001.pdf]
