# Supplementary material for: Agreement between gastrointestinal panel testing and standard microbiology methods for detecting pathogens in suspected infectious gastroenteritis: Test evaluation and meta-analysis in the absence of a reference standard
Source: PLoS One. 2017 Mar 2;12(3):e0173196. doi: 10.1371/journal.pone.0173196 (PMC5333893; doi:10.1371/journal.pone.0173196)
Supplement: S1 File — (PDF) [file pone.0173196.s002.pdf]

S1 File. Search strategy for clinical effectiveness review

Medline and Embase searches

1. exp Gastroenteritis/
2. exp *Diarrhea/
3. exp *Feces/
4. exp *Gastroenteritis/
5. exp *Gastrointestinal Diseases/
6. (gastrointestin* or stool* or enteric* or feces or faeces or diarrh?ea).tw.
7. 1 or 2 or 3 or 4 or 5 or 6
8. Multiplex Polymerase Chain Reaction/
9. (xtag or Luminex or Filmarray or biofire).tw.
10. ("Faecal Pathogens B" or "Faecal Panel B" or ausdiagnostics).tw.
11. (multiplex* adj4 (PCR or polymerase chain reaction or assay* or panel* or test*)).tw.
12. (gastrointestinal pathogen panel or gastrointestinal infection panel).tw.
13. 8 or 9 or 10 or 11 or 12
14. 7 and 13

Ovid MEDLINE(R) 1946 to November Week 3 2015 - 1424 downloaded

Ovid MEDLINE(R) In-Process & Other Non-Indexed Citations December 31, 2015 - 138 downloaded

Ovid Embase1980 to 2015 Week 52 - 1803 downloaded

Web of Science Core Collection search strategy – 1980 to 31/12/2015

#4	#3 OR #2 OR #1<br>*DocType=All document types; Language=All languages;*
#3	**TITLE:** (xtag or Luminex or Filmarray or biofire or "Faecal Pathogens B" or "Faecal Panel B" or ausdiagnostics) *AND***TOPIC:** (gastro*)<br>*DocType=All document types; Language=All languages;*
#2	TS=("gastrointestinal pathogen panel" or "gastrointestinal infection panel")<br>*DocType=All document types; Language=All languages;*
#1	**TITLE:** (multiplex* and (PCR or polymerase chain reaction or assay* or panel* or test*)) *AND***TOPIC:** (gastrointestin*)<br>*DocType=All document types; Language=All languages;*

98 downloaded

Other searches

- Searched all sections of the Cochrane Database of Systematic Reviews (including the NHS Economic Evaluation Database : Issue 2 of 4, April 2015) – no additional studies found

Auto-alerts

Weekly auto-alerts were run in Ovid Medline, Ovid Embase and PubMed from 1/1/2016 to 31/4/2016 to check for any new studies added subsequent to the main searches.

Search strategies for ongoing studies

- NIH ClinicalTrials.gov (http://www.clinicaltrials.gov/) - 1 downloaded
- Current Controlled Trials (http://www.controlled-trials.com)
- WHO International Clinical Trials Registry Platform (ICTRP) (http://www.who.int/ictrp/en/)
- UK Clinical Trials Gateway (UKCTG)http://www.nihr.ac.uk/research/uk-clinical-trials-gateway.htm
