# Supplementary material for: Agreement between gastrointestinal panel testing and standard microbiology methods for detecting pathogens in suspected infectious gastroenteritis: Test evaluation and meta-analysis in the absence of a reference standard
Source: PLoS One. 2017 Mar 2;12(3):e0173196. doi: 10.1371/journal.pone.0173196 (PMC5333893; doi:10.1371/journal.pone.0173196)
Supplement: S1 Table — (PDF) [file pone.0173196.s003.pdf]

S1 Table. Overview of study characteristics of included studies

Study reference	GPP test	Population / sample size / characteristics	Study design	Agreement outcome reported
Buss 2015[7]<br>USA<br>4 centres	FilmArray	N=1556<br>Hospitalised: 164 (10.5%)<br>ER: 42 (2.7%)<br>Outpatients: 1,350 (86.8%)<br>Adults above 21 years: 584 (38%)<br>Children 0-5 years: 539 (35%)	**Study set up:**<br>Culture was set up as part of routine clinical testing at 4 testing sites using their standard procedure, submitting physicians may have ordered testing in addition to stool culture; however, the results of such routine testing were not collected or utilized in this study for comparator analysis. FilmArray was done at study sites<br>**Comparator:**<br>Bacteria: specimens were tested using stool culture<br>Viruses / parasites / *Clostridium difficile* toxins / STEC / ETEC / EPEC / EAEC: Real-time PCR (two independent well-validated assays for each analyte different from the FilmArray GI Panel targets if possible) and sequence analysis were performed by BioFire personnel. Comparator regarded positive if both tests positive if discrepant both PCRs repeated<br>All samples received all comparator tests with two exceptions regarding interpretation of results: In order to follow the same algorithm as FilmArray utilizes, EPEC results were classed as NA if they were STEC positive and E. coli O157 results were only considered if the STEC test was positive<br>**GPP:**<br>IUO version therefore including results for *Aeromonas*, all samples tested with FimArray at study sites within 4 days of sampling, samples shipped frozen on a weekly basis<br>**Verification:**<br>Discordant samples tested blinded by BioFire, PCR and sequencing using different targets to comparator method and FilmArray, or using enhanced methods (additional PCR cycles and replicate samples) or using benchtop version of FilmArray (FilmArray primers in conventional real-time PCR).	Positive percent agreement and negative percent agreement instead of sensitivity and specificity to indicate that a non-gold-standard assay (e.g., PCR) was used for the comparator analysis.

Study reference	GPP test	Population / sample size / characteristics	Study design	Agreement outcome reported
Claas 2013[8]<br>Netherlands, USA, Canada, UK<br>4 Centres	xTAG	N=901	**Study set up:**<br>Each participating laboratory analysed specimens according to the routine diagnostic algorithm in place at that site, and as ordered by the referring physician. All samples shipped to Luminex for GPP testing<br>**Comparator:**<br>As ordered by referring physician at 4 study sites following their routine diagnostic algorithms<br>Bacteria: culture according to standard procedures at 4 sites<br>Bacterial toxins: EIA (North America)<br>Parasites: microscopy or EIA (North America), microscopy or PCR (Europe)<br>Viruses: PCR (Europe only)<br>**GPP:**<br>xTAG performed by Luminex<br>**Verification:**<br>A subset of samples was assessed by conventional PCR and bidirectional sequencing using validated primers targeting genomic regions distinct from those of the xTAG GPP, site of testing not reported	Sensitivity and specificity

Study reference	GPP test	Population / sample size / characteristics	Study design	Agreement outcome reported
Coste 2013[9]<br>France<br>1 Centre	xTAG	54 samples of 49 adult kidney transplant recipients<br>Median age: 51 years<br>Range: 18-78 years<br>Male: 30/49 (61%)<br>Median post transplantation term: 6.3 years (range 3 days to 24.2 years)<br>Immunocompromised: 49/49 (100%)	**Study set up:**<br>Stool samples were taken from each study participant at the time of the severe diarrhoea episode, parasitological tests were performed again on a second sample taken 72 h after inclusion to deal with the shedding of intestinal protozoa. Routine microbiology testing at study site, samples stored and retrospectively tested by 7 GPP assays at the study site<br>**Comparator:**<br>Bacteria: Culture<br>*C. difficile*: EIA and culture<br>Parasites: microscopy<br>Viruses: rapid antigen detection tests<br>Assumed that all patients received all mentioned conventional tests<br>**GPP:**<br>xTAG on stored frozen samples at study site<br>**Verification:**<br>Stool samples positive for one of the enteric viruses and for *Campylobacter* spp. As well as 23 negative samples by GPP were sent to a National Reference Centre for confirmation and typing.	Sensitivity, specificity, positive predictive value (PPV), and negative predictive value (NPV) for norovirus and Campylobacter

Study reference	GPP test	Population / sample size / characteristics	Study design	Agreement outcome reported
Deng 2015[10]<br>China<br>1 centre	xTAG	290 stool specimens of 290 diarrhoeal patients<br>Inpatients: 70/290 (24%)<br>Outpatients: 220/290 (76%)<br>Male: 186/290 (64%)<br>Median age: 25 months<br>Age range: 11 days to 83 years	**Study set up:**<br>Stool specimens prospectively collected and submitted to clinical laboratory. All stool samples were tested for all 17 pathogens using routine methods using standard procedures. All samples received singleplex PCR and sequencing for *C. difficile*. All samples received xTAG.<br>**Comparator:**<br>Disregarding what physician ordered all samples were tested for 17 pathogens at clinical laboratory<br>Bacteria: Culture confirmed by gene sequencing, mass spectrometry or serotyping<br>Viruses: Immunochromatography<br>Norovirus: Real-time reverse transcription PCR<br>Parasites: microscopy<br>*C. difficile* not tested<br>**GPP:**<br>xTAG performed at study site on all samples<br>**Verification:**<br>Samples discordant between the routine tests and xTAG were tested by singleplex PCR and sequencing using primers from published literature which were synthesised by Sangon Biotech (assumed to be different to xTAG primers).	Agreement using kappa coefficient test, sensitivity and specificity

Study reference	GPP test	Population / sample size / characteristics	Study design	Agreement outcome reported
Duong 2016[11]<br>Vietnam<br>>3 Centres	xTAG	479 patients hospitalised with diarrhoeal disease<br>Adults age: median 50 years (IQR 33-64)<br>Children age: 16.5 months (IQR 6.7-20 months)<br>Adult male: 36/92 (39%)<br>Children male: 221/387 (57%)	**Study set up:**<br>Fresh stool was stored at 4°C at sites and transported to the central study microbiology laboratory within 24 hours. The specimens were tested using microbiological culture and real-time PCR then stored at -80°C for xTAG testing at study laboratory. All samples were tested for *Shigella,Salmonella*, *Campylobacter* by culture and PCR and all were tested for adenovirus, norovirus, and rotavirus by PCR, culture and PCR were evaluated separately<br>**Comparator:**<br>All samples received all tests for pathogens considered in the study<br>Bacteria: Culture and PCR<br>Viruses: PCR<br>**GPP:**<br>xTAG of all samples at study laboratory<br>**Verification:**<br>No verification undertaken, sensitivity and specificity were calculated for culture as gold standard and for PCR as gold standard	Sensitivity and specificity

Study reference	GPP test	Population / sample size / characteristics	Study design	Agreement outcome reported
FDA 2012[12]<br>USA, Canada<br>6 centres	xTAG	N=1534 patients (including outbreak samples)<br>Male: 632 (44.9%)<br>12-21 years: n=51 (3.6%),<br>21-65 years: n=879 (62.5%),<br>>65 years: n=426 (30.3%)<br><u>Subject status</u><br>Outpatients: n=421 (29.9%), n=hospitalised (57.1%),<br>emergency department: n=118 (8.4%),<br>long term care facility: n=18 (1.3%),<br>not determined: n=46 (3.3%),<br><u>Immune status</u><br>Immuno-compromised n=493 (35%), immunocompetent: n=758 (54%),<br>not determined: 156 (11.12%)	**Study set up:**<br>Prospective clinical specimens were submitted fresh to the sites and were processed according to their routine algorithm.<br>**Comparator:**<br>Conventional methods were ordered by the referring physician following routine methods at sites, but these were not included in analysis. Comparator methods for all prospective samples were undertaken in central reference laboratories. In the event that comparator results were not available for all targets on a given specimen, then the specimen in question was excluded from performance calculations of xTAG.<br>*Salmonella*, *Shigella*, *E.coli*: Culture<br>*Campylobacter*: Culture and PCR / sequencing assay for *Campylobacter*+ samples<br>STEC: Broth enrichment and Immunocard assay<br>ETEC: 4 PCR/sequencing assays<br>*C. difficile*: Cytotoxity assay<br>*Cryptosporidium*, *Giardia*: microscopy<br>Norovirus: real-time PCR and conventional PCR with bidirectional sequencing<br>Rotavirus: EIA and PCR/sequencing assay<br>**GPP:**<br>xTAG on all samples at 6 study sites. 14 pathogens (excluding<br>Yersinia)<br>**Verification:**<br>Discrepant results between the xTAG GPP and the reference methods were evaluated using analytically validated PCR/sequencing assays (bi-directional sequencing analysis) using primers not covered by the xTAG GPP kit primers or FDA cleared molecular assays (i.e., for C. difficile Toxin), central laboratories undertook verification	Sensitivity and specificity (positive and negative percent agreements)

Study reference	GPP test	Population / sample size / characteristics	Study design	Agreement outcome reported
Gu 2015[13]<br>USA<br>1 centre	xTAG and FilmArray	436 samples of 199 paediatric oncology patients	**Study set up:**<br>After routine clinical testing, remnant samples were stored at -80°C for 30 to 44 months before testing with GPP assays.<br>All samples were tested with xTAG and FilmArray.<br>**Comparator:**<br>Standard-of-care testing at study site on clinical suspicion and subsequent ordering of routine test (172 samples), in addition to routine tests astro-, noro- and sapovirus PCR was tested on all samples, standard care tests plus multiplex PCRs for 3 additional viruses made up comparator<br>Viruses: multiplex PCR (adenovirus multiplex PCR detected all serotypes not limited to enteric adenovirus)<br>Rotavirus: EIA<br>Bacteria: Culture<br>*C. difficile*: PCR<br>Parasites: EIA<br>**GPP:**<br>All samples received xTAG and FilmArray at study site<br>**Verification:**<br>No verification undertaken, agreement was reported either out of 172 patients having received conventional methods or out of 199 patients having received comparator.	Sensitivity and Specificity using in house methods as reference standard

Study reference	GPP test	Population / sample size / characteristics	Study design	Agreement outcome reported
Halligan 2014[14]<br>UK<br>1 centre	xTAG	N=2187<br>Hospital-associated cases: 1201/2187 (55%)<br>Community associated cases: 986/2187 (45%)	**Study set up:**<br>Clinicians were advised to investigate all cases of diarrhoea selecting tests from a menu including bacteria (*Campylobacter*, *Salmonella*, *Shigella* and *Escherichia coli* O157 with *Vibrio* and *Yersinia* species available upon specific request), norovirus (samples received from children aged 5 years and under are automatically tested for rotavirus and faecal adenovirus in addition), parasites and *Clostridium difficile* testing that is either performed on request or automatically on samples from patients over the age of 65 years. Conventional testing was performed 7 days per week. Clinicians were advised not to send samples for bacterial culture if the onset of symptoms was >3 days following hospital admission. Samples were stored at 4°C until testing was complete. Clinicians were unable to request a GPP test directly, instead whenever a request for conventional testing was received, a GPP request was included.<br>**Comparator:**<br>Testing according to Physician’s request at study centre.<br>Bacteria: Culture<br>*C. difficile*: EIA followed by PCR<br>Viruses: EIA<br>Parasites: Microscopy<br>**GPP:**<br>All samples received xTAG at study centre<br>Samples were batched for DNA extraction at 4pm Monday to Thursday, further analysis commenced the following morning with results available at 3pm. Alternative run on Friday 10am for late evening or Saturday reporting<br>**Verification:**<br>Assumption that xTAG results are correct, no verification undertaken	Agreement and kappa, sensitivity and specificity were not calculated because of the lack of a comparable reference standard or resolving assay

**Study reference**	**GPP test**	**Population / sample size / characteristics**	**Study design**	**Agreement outcome reported**
Mengelle 2013[15]<br>France<br>1 centre	xTAG	440 samples of 329 diarrhoeic patients<br>Immunosuppressed hospitalized patients:<br>1) 102 adult organ transplant recipients (mean age, 50.6;<br>median, 56; range, 17–75)<br>2) 50 immunocompromised children (mean age, 5; median, 7; range, 0–14)<br>3) 56 children attending the neonatal unit (aged under 1 year)<br>121 children attending the emergency unit (mean age, 2.80; median, 9; range, 0–16) were considered to be outpatients	**Study set up:**<br>Prospectively collected stool samples, C. diff and Escherichia coli pathovars tested in all samples from children, but only in those from certain adults: post-antibiotherapy diarrhoea and nosocomial outbreaks for toxigenic C. diff, or an epidemiological infection for STEC. Conventional and GPP undertaken at study site<br>**Comparator:**<br>All had at least one conventional test according to physician's judgment<br>Bacteria: culture<br>*C. difficile*: Immunochromatographic test<br>Viruses: rapid immunochromatographic test<br>Parasites: microscopy<br>**GPP:**<br>xTAG on all samples at study site<br>**Verification:**<br>Samples showing discrepant adenovirus results were tested with an in-house real-time PCR	Proportions of positives by conventional and GPP method and McNemar's test

Study reference	GPP test	Population / sample size / characteristics	Study design	Agreement outcome reported
Pankhurst 2014[16]<br>UK<br>1 centre	xTAG	N=839	**Study set up:**<br>A retrospective study of fixed numbers of samples positive for *C. difficile*, *Campylobacter* spp., *Salmonella* spp. and norovirus plus samples negative for all these pathogens. All samples collected were initially sent to the service microbiology laboratory for faecal culture and/or *C. difficile* toxin testing by hospital-based doctors or GPs as a result of a suspected enteric infection. xTAG was undertaken at the study site on all samples.<br>**Comparator:**<br>Initial diagnosis of the target faecal pathogens was performed in accordance with Public Health England guidelines in the service microbiology laboratory for faecal culture and/or *C. difficile* toxin testing requested by hospital-based doctors or GPs. All 839 patients had results for all 4 pathogens<br>*C. difficile*: EIA testing for toxins A and B with subsequent serological and sensitivity testing<br>*Salmonella* and *Campylobacter*: culture<br>Norovirus: qPCR<br>**GPP:**<br>All samples were tested for all 15 pathogens (xTAG) but only comparison to conventional testing for 4 most common: *C. difficile*, *Campylobacter* spp., *Salmonella* spp. and norovirus, different laboratory to conventional testing<br>**Verification:**<br>Unexpectedly positive or negative for target organisms were retested in duplicate using qPCR assays, same lab not blinded samples.<br>Positive on standard reference microbiology but negative on xTAG and confirmed negative on qPCR were considered negative, and samples negative on standard reference microbiology but positive on xTAG and confirmed positive on qPCR were considered positive.	Sensitivity and Specificity

Study reference	GPP test	Population / sample size / characteristics	Study design	Agreement outcome reported
GPP gastrointestinal panel, FDA Food and Drug Administration; EIA enzyme immunoassay, PCR polymerase chain reaction, EAEC Enteroaggregative *Escherichia coli*, *C. difficile Clostridium difficile*, STEC Shiga toxin-producing *Escherichia coli*, ETEC Enterotoxigenic *Escherichia coli*, *E.coli Escherichia coli*, qPCR quantitative polymerase chain reaction, EPEC Enteropathogenic *Escherichia coli*, *E. coli* O157 *Escherichia coli* O157, spp species, GP general practitioner; IC immunocompromised, ER emergency room				
