# Supplementary material for: Agreement between gastrointestinal panel testing and standard microbiology methods for detecting pathogens in suspected infectious gastroenteritis: Test evaluation and meta-analysis in the absence of a reference standard
Source: PLoS One. 2017 Mar 2;12(3):e0173196. doi: 10.1371/journal.pone.0173196 (PMC5333893; doi:10.1371/journal.pone.0173196)
Supplement: S2 Table — (PDF) [file pone.0173196.s004.pdf]

S2 Table. Judgement of risk of bias and applicability of included studies

Study	RISK OF BIAS					APPLICABILITY CONCERNS			
	Patient Selection	Index Test	Comparator	Reference Standard	Flow and Timing	Patient Selection	Index Test	Comparator	Reference Standard
Buss 2015[7]	unclear	low	high	high	high	high	low	high	high
Claas 2013[8]	unclear	low	high	high	high	low	low	low	high
Coste 2013[9]	low	low	high	high	high	high	low	high	high
Deng 2015[10]	unclear	low	high	high	Low	high	low	high	high
Duong 2016[11]	unclear	low	high	NA	high	high	low	high	NA
FDA 2012[12]	unclear	low	high	high	high	low	low	high	high
Gu 2015[13]	high	low	high	NA	high	high	low	low	NA
Halligan 2014[14]	high	low	high	NA	high	low	low	low	NA
Mengelle 2013[15]	high	unclear	high	high	high	low	unclear	low	high
Pankhurst 2014[16]	unclear	low	high	high	low	low	low	high	high

NA – not applicable (the study did not use any methods to verify discordant outcomes)
