# Supplementary material for: Agreement between gastrointestinal panel testing and standard microbiology methods for detecting pathogens in suspected infectious gastroenteritis: Test evaluation and meta-analysis in the absence of a reference standard
Source: PLoS One. 2017 Mar 2;12(3):e0173196. doi: 10.1371/journal.pone.0173196 (PMC5333893; doi:10.1371/journal.pone.0173196)
Supplement: S3 Table — (PDF) [file pone.0173196.s005.pdf]

S3 Tables. 2x2 data by pathogen and GPP test

2x2 data in the tables below are reported in the following format:

	Conventional test +	Conventional test -
GPP +	a (+/+)	b (-/+)
GPP -	c (+/-)	d (-/-)

Viruses

Adenovirus

**Study**	**a**	**b**	**c**	**d**	**Total**
Claas 2013[8]	4	4	16	628	652
Deng 2015[10]	3	0	2	285	290
Duong 2016[11]	23	5	2	449	479
FDA 2012[12]	4	13	1	1154	1172
Gu 2015[13]	3	2	20	110	135
Halligan 2014[14]	6	2	0	181	189
Mengelle 2013[15]	0	2	5	396	403

a – conventional test positive / GPP test positive, b - conventional test negative / GPP test positive, c - conventional test positive / GPP test negative, d - conventional test negative / GPP test negative; n/e – not estimable

Norovirus

**Study**	**a**	**b**	**c**	**d**	**Total**
Claas 2013[8] G1	9	0	0	642	651
Claas 2013[8] G2	62	14	5	570	651
Deng 2015[10] G1	8	0	0	282	290
Deng 2015[10] G2	37	3	2	248	290
Duong 2016[11] G1	15	5	2	457	479
Duong 2016[11] G2	85	4	3	387	479
Gu* 2015[13]	29	9	2	159	199
Halligan 2014[14]	66	79	14	1284	1443
Mengelle 2013[15]	0	17	1	250	268
Pankhurst 2014[16]	183	16	16	624	839
FDA 2012[12]	74	98	4	1023	1199
Coste 2013[9]	0	14	0	40	54

a – conventional test positive / GPP test positive, b - conventional test negative / GPP test positive, c - conventional test positive / GPP test negative, d - conventional test negative / GPP test negative; n/e – not estimable

Rotavirus

Study	a	b	c	d	Total
Claas 2013[8]	18	1	0	633	652
Deng 2015[10]	61	6	1	222	290
Duong 2016[11]	117	4	9	349	479
Gu 2015[13]	1	0	0	109	110
Halligan 2014[14]	13	6	0	158	177
Mengelle 2013[15]	61	9	2	332	404
FDA 2012[12]	2	2	0	1162	1166
Coste 2013[9]	2	0	0	52	54

a – conventional test positive / GPP test positive, b - conventional test negative / GPP test positive, c - conventional test positive / GPP test negative, d - conventional test negative / GPP test negative; n/e – not estimable

Bacteria

C. difficile

Study	a	b	c	d	Total
Claas 2013[8]	30	11	1	343	385
Gu 2015[13]	44	8	4	107	163
Halligan 2014[14]	121	10	6	1175	1312
Mengelle 2013[15]	5	8	0	331	344
Pankhurst 2014[16]	195	19	4	621	839
FDA 2012[12]	107	105	7	922	1141
Coste 2013[9]	1	0	0	53	54

a – conventional test positive / GPP test positive, b - conventional test negative / GPP test positive, c - conventional test positive / GPP test negative, d - conventional test negative / GPP test negative; n/e – not estimable

Campylobacter

Study	a	b	c	d	Total

Claas 2013[8]	111	15	3	382	511
Deng 2015[10]	20	16	0	254	290
Duong 2016[11] PCR	59	3	6	411	479
Duong 2016[11] Culture	27	35	3	414	479
Gu 2015[13]	0	0	0	112	112
Halligan 2014[14]	23	37	0	1336	1396
Mengelle 2013[15]	1	12	0	368	381
Pankhurst 2014[16]	110	11	7	711	839
FDA 2012[12]	3	21	0	1155	1179
Coste 2013[9]	2	12	0	40	54

a – conventional test positive / GPP test positive, b - conventional test negative / GPP test positive, c - conventional test positive / GPP test negative, d - conventional test negative / GPP test negative; n/e – not estimable

E.coli O157

Study	a	b	c	d	Total
Claas 2013[8]	15	5	1	407	428
Deng 2015[10]	1	2	0	287	290
Gu* 2015[13]	0	1	0	111	112
Halligan 2014[14]	3	2	0	1391	1396
Mengelle 2013[15]	0	1	0	265	266
FDA 2012[12]	2	9	0	1158	1169

a – conventional test positive / GPP test positive, b - conventional test negative / GPP test positive, c - conventional test positive / GPP test negative, d - conventional test negative / GPP test negative
*study did not separate out E.coli O157, EAEC, EPEC, ETEC, STEC; n/e – not estimable

ETEC

Study	a	b	c	d	Total
Claas 2013[8]	0	7	0	68	75
Deng 2015[10]	1	4	0	285	290
Mengelle 2013[15]	0	0	0	266	266
FDA 2012[12]	2	4	6	1156	1168

a – conventional test positive / GPP test positive, b - conventional test negative / GPP test positive, c - conventional test positive / GPP test negative, d - conventional test negative / GPP test negative; n/e – not estimable

Salmonella

Study	a	b	c	d	Total
Claas 2013[8]	62	6	13	408	489
Deng 2015[10]	25	6	5	254	290
Duong 2016[11] PCR	84	128	9	258	479
Duong 2016[11] Culture	38	172	2	267	479
Gu 2015[13]	1	3	0	108	112
Halligan 2014[14]	11	36	0	1349	1396
Mengelle 2013[15]	7	14	2	356	379
Pankhurst 2014[16]	15	9	18	797	839
FDA 2012[12]	10	18	0	1143	1171
Coste 2013[9]	0	1	0	53	54

a – conventional test positive / GPP test positive, b - conventional test negative / GPP test positive, c - conventional test positive / GPP test negative, d - conventional test negative / GPP test negative; n/e – not estimable

Shigella

Study	a	b	c	d	Total
Claas 2013[8]	40	13	0	452	505
Deng 2015[10]	3	1	0	286	290
Duong 2016[11] PCR	86	6	4	383	479
Duong 2016[11] Culture	40	51	0	388	479
Gu 2015[13]	1	0	0	111	112
Halligan 2014[14]	3	11	0	1382	1396
Mengelle 2013[15]	1	0	0	378	379
FDA 2012[12]	2	17	0	1154	1173

a – conventional test positive / GPP test positive, b - conventional test negative / GPP test positive, c - conventional test positive / GPP test negative, d - conventional test negative / GPP test negative; n/e – not estimable

STEC

Study	a	b	c	d	Total
Claas 2013[8]	8	1	0	162	171
Mengelle 2013[15]	5	2	0	258	265
FDA 2012[12]	1	16	0	1153	1170
Coste 2013[9]	0	2	1	51	54

a – conventional test positive / GPP test positive, b - conventional test negative / GPP test positive, c - conventional test positive / GPP test negative, d - conventional test negative / GPP test negative; n/e – not estimable

Vibrio cholera

Study	a	b	c	d	Total
Claas 2013[8]	0	0	1	194	195
Deng 2015[10]	0	0	0	290	290
FDA 2012[12]	0	1	0	1166	1167
Mengelle 2013[15]	0	0	0	379	379

a – conventional test positive / GPP test positive, b - conventional test negative / GPP test positive, c - conventional test positive / GPP test negative, d - conventional test negative / GPP test negative; n/e – not estimable

Yersinia enterocolitica

Study	a	b	c	d	Total
Claas 2013[8]	0	0	0	366	366
Deng 2015[10]	1	0	0	289	290
Gu 2015[13]	0	0	0	38	38
Mengelle 2013[15]	0	0	0	379	379

a – conventional test positive / GPP test positive, b - conventional test negative / GPP test positive, c - conventional test positive / GPP test negative, d - conventional test negative / GPP test negative; n/e – not estimable

Parasites

Cryptosporidium

Study	a	b	c	d	Total
Claas 2013[8]	32	0	3	833	868
Gu 2015[13]	0	0	0	35	35

Halligan 2014[14]	1	6	0	229	236
Mengelle 2013[15]	0	1	0	117	118
FDA 2012[12]	12	53	1	1131	1197

a – conventional test positive / GPP test positive, b - conventional test negative / GPP test positive, c - conventional test positive / GPP test negative, d - conventional test negative / GPP test negative; n/e – not estimable

Entamoeba histolytica

**Study**	**a**	**b**	**c**	**d**	**Total**
Claas 2013[8]	6	6	0	845	857
Deng 2015[10]	0	1	0	289	290
Halligan 2014[14]	0	9	1	226	236
FDA 2012[12]	0	19	0	1149	1168
Mengelle 2013[15]	0	0	0	285	285

a – conventional test positive / GPP test positive, b - conventional test negative / GPP test positive, c - conventional test positive / GPP test negative, d - conventional test negative / GPP test negative; n/e – not estimable

Giardia

**Study**	**a**	**b**	**c**	**d**	**Total**
Claas 2013[8]	26	13	0	829	868
Deng 2015[10]	0	0	0	290	290
Gu 2015[13]	0	0	0	35	35
Halligan 2014[14]	1	17	0	218	236
Mengelle 2013[15]	0	0	0	118	118
FDA 2012[12]	4	39	0	1132	1175
Coste 2013[9]	1	0	0	53	54

a – conventional test positive / GPP test positive, b - conventional test negative / GPP test positive, c - conventional test positive / GPP test negative, d - conventional test negative / GPP test negative; n/e – not estimable

FilmArray BioFire

Viruses

Adenovirus

**Study**	**a**	**b**	**c**	**d**	**Total**

Buss 2015[7]	42	13	2	1499	1556
Gu 2015[13]	3	2	20	110	135

a – conventional test positive / GPP test positive, b - conventional test negative / GPP test positive, c - conventional test positive / GPP test negative, d - conventional test negative / GPP test negative; n/e – not estimable

Astrovirus

**Study**	**a**	**b**	**c**	**d**	**Total**
Buss 2015[7]	7	1	0	1548	1556
Gu 2015[13]	4	0	6	189	199

a – conventional test positive / GPP test positive, b - conventional test negative / GPP test positive, c - conventional test positive / GPP test negative, d - conventional test negative / GPP test negative; n/e – not estimable

Norovirus

**Study**	**a**	**b**	**c**	**d**	**Total**
Buss 2015[7]	52	18	3	1483	1556
Gu 2015[13]	28	0	3	168	199

a – conventional test positive / GPP test positive, b - conventional test negative / GPP test positive, c - conventional test positive / GPP test negative, d - conventional test negative / GPP test negative; n/e – not estimable

Rotavirus

**Study**	**a**	**b**	**c**	**d**	**Total**
Buss 2015[7]	6	12	0	1538	1556
Gu 2015[13]	1	0	0	109	110

a – conventional test positive / GPP test positive, b - conventional test negative / GPP test positive, c - conventional test positive / GPP test negative, d - conventional test negative / GPP test negative; a/a+b+c – positive agreement; d/b+c+d – negative agreement; n/e – not estimable

Sapovirus

**Study**	**a**	**b**	**c**	**d**	**Total**
Buss 2015[7]	46	13	0	1497	1556

Gu 2015[13]	5	0	2	192	199

a – conventional test positive / GPP test positive, b - conventional test negative / GPP test positive, c - conventional test positive / GPP test negative, d - conventional test negative / GPP test negative; n/e – not estimable

Bacteria

C. difficile

Study	a	b	c	d	Total
Buss 2015[7]	163	41	2	1350	1556
Gu 2015[13]	39	5	9	110	163

a – conventional test positive / GPP test positive, b - conventional test negative / GPP test positive, c - conventional test positive / GPP test negative, d - conventional test negative / GPP test negative; n/e – not estimable

Campylobacter

Study	a	b	c	d	Total
Buss 2015[7]	34	24	1	1497	1556
Gu 2015[13]	0	0	0	112	112

a – conventional test positive / GPP test positive, b - conventional test negative / GPP test positive, c - conventional test positive / GPP test negative, d - conventional test negative / GPP test negative; n/e – not estimable

Shigella

Study	a	b	c	d	Total
Buss 2015[7]	47	2	2	1505	1556
Gu 2015[13]	1	0	0	111	112

a – conventional test positive / GPP test positive, b - conventional test negative / GPP test positive, c - conventional test positive / GPP test negative, d - conventional test negative / GPP test negative; n/e – not estimable

STEC

Study	a	b	c	d	Total
Buss 2015[7]	33	5	0	1518	1556

a – conventional test positive / GPP test positive, b - conventional test negative / GPP test positive, c - conventional test positive / GPP test negative, d - conventional test negative / GPP test negative; n/e – not estimable

Vibrio (parahaemolyticus, vulnificus and cholerae)

Study	a	b	c	d	Total
Buss 2015[7]	0	2	0	1554	1556
Gu 2015[13]	0	1	0	111	112

a – conventional test positive / GPP test positive, b - conventional test negative / GPP test positive, c - conventional test positive / GPP test negative, d - conventional test negative / GPP test negative; n/e – not estimable

Vibrio cholera

Study	a	b	c	d	Total
Buss 2015[7]	0	1	0	1555	1556

a – conventional test positive / GPP test positive, b - conventional test negative / GPP test positive, c - conventional test positive / GPP test negative, d - conventional test negative / GPP test negative; n/e – not estimable

Yersinia enterocolitica

Study	a	b	c	d	Total
Buss 2015[7]	1	0	0	1555	1556
Gu 2015[13]	0	0	0	38	38

a – conventional test positive / GPP test positive, b - conventional test negative / GPP test positive, c - conventional test positive / GPP test negative, d - conventional test negative / GPP test negative; an/e – not estimable

E.coli O157

Study	a	b	c	d	Total
Buss 2015[7]	3	1	0	34	38
Gu* 2015[13]	0	4	0	108	112

a – conventional test positive / GPP test positive, b - conventional test negative / GPP test positive, c - conventional test positive / GPP test negative, d - conventional test negative / GPP test negative; n/e – not estimable

EAEC

**Study**	**a**	**b**	**c**	**d**	**Total**
Buss 2015[7]	82	27	1	1446	1556

a – conventional test positive / GPP test positive, b - conventional test negative / GPP test positive, c - conventional test positive / GPP test negative, d - conventional test negative / GPP test negative; n/e – not estimable

EPEC

**Study**	**a**	**b**	**c**	**d**	**Total**
Buss 2015[7]	314	34	3	1167	1518

a – conventional test positive / GPP test positive, b - conventional test negative / GPP test positive, c - conventional test positive / GPP test negative, d - conventional test negative / GPP test negative; n/e – not estimable

ETEC

**Study**	**a**	**b**	**c**	**d**	**Total**
Buss 2015[7]	22	9	0	1525	1556

a – conventional test positive / GPP test positive, b - conventional test negative / GPP test positive, c - conventional test positive / GPP test negative, d - conventional test negative / GPP test negative; n/e – not estimable

Plesiomonas shigelloides

**Study**	**a**	**b**	**c**	**d**	**Total**
Buss 2015[7]	3	15	0	1538	1556

a – conventional test positive / GPP test positive, b - conventional test negative / GPP test positive, c - conventional test positive / GPP test negative, d - conventional test negative / GPP test negative; n/e – not estimable

Salmonella

Study	a	b	c	d	Total
Buss 2015[7]	31	6	0	1519	1556
Gu 2015[13]	1	0	0	111	112

a – conventional test positive / GPP test positive, b - conventional test negative / GPP test positive, c - conventional test positive / GPP test negative, d - conventional test negative / GPP test negative; n/e – not estimable

Parasites

Entamoeba histolytica

Study	a	b	c	d	Total
Buss 2015[7]	0	0	0	1556	1556

a – conventional test positive / GPP test positive, b - conventional test negative / GPP test positive, c - conventional test positive / GPP test negative, d - conventional test negative / GPP test negative; n/e – not estimable

Cryptosporidium

Study	a	b	c	d	Total
Buss 2015[7]	18	6	0	1532	1556
Gu 2015[13]	0	0	0	35	35

a – conventional test positive / GPP test positive, b - conventional test negative / GPP test positive, c - conventional test positive / GPP test negative, d - conventional test negative / GPP test negative; n/e – not estimable

Cyclospora cayetanensis

Study	a	b	c	d	Total
Buss 2015[7]	19	0	0	1537	1556

a – conventional test positive / GPP test positive, b - conventional test negative / GPP test positive, c - conventional test positive / GPP test negative, d - conventional test negative / GPP test negative; n/e – not estimable

Giardia

Study	a	b	c	d	Total
Buss 2015[7]	20	7	0	1529	1556

Gu 2015[13]	0	0	0	35	35

a – conventional test positive / GPP test positive, b - conventional test negative / GPP test positive, c - conventional test positive / GPP test negative, d - conventional test negative / GPP test negative; n/e – not estimable
