# Supplementary material for: Agreement between gastrointestinal panel testing and standard microbiology methods for detecting pathogens in suspected infectious gastroenteritis: Test evaluation and meta-analysis in the absence of a reference standard
Source: PLoS One. 2017 Mar 2;12(3):e0173196. doi: 10.1371/journal.pone.0173196 (PMC5333893; doi:10.1371/journal.pone.0173196)
Supplement: S4 Table — (PDF) [file pone.0173196.s006.pdf]

S4 Table. Outcomes of verification of discordant results in 4 studies reporting outcomes of verification by pathogen using a third method

Study (GPP test)	Pathogen	Comparator	Verification method (judgement of bias)	Reported results of verification*
Buss 2015[7] (FilmArray)	Adenovirus 40/41	2x PCR	ECP (biased), BTFA (biased)	Comparator + / Comparator –<br>GPP +: 42 / 13 (verified 11+/0, 2 remain inconclusive**)<br>GPP -: 2 (verified 1+/0, 1 remains inconclusive) / 1499
	C. difficile toxin A/B	2x PCR	ECP (biased), BTFA (biased)	Comparator + / Comparator –<br>GPP +: 163 / 41 (verified 41+/0)<br>GPP -: 2 (verified 1+/0 and 1 remains inconclusive) / 1350
	Campylobacter	Culture	cadF and gyrA PCR (biased), BTFA (biased)	Comparator + / Comparator –<br>GPP +: 34 / 24 (verified 19+/0, 5 remain inconclusive)<br>GPP -: 1 (verified 1+/0) / 1497
	Cryptosporidium	2x PCR	ECP, 18S rRNA gene PCR (biased), BTFA (biased)	Comparator + / Comparator –<br>GPP +: 18 / 6 (verified 6+/0)<br>GPP -: 0 / 1532
	E. coli O157	Culture (STEC positive only)	rfbE PCR (biased)	Comparator + / Comparator –<br>GPP +: 3 / 1 (verified 1+/0)<br>GPP -: 0 / 34
	ETEC	3x PCR	ECP (biased), BTFA (biased)	Comparator + / Comparator –<br>GPP +: 22 / 9 (verified 6+/3-)<br>GPP -: 0 / 1525
	Giardia	2x PCR (one using published primers)	ECP (biased), BTFA (biased)	Comparator + / Comparator –<br>GPP +: 20 / 7 (verified 4+/2-, 2 remain inconclusive)<br>GPP -: 0 / 1529
	Norovirus	PCR (using published primers)	PCR with multiple primer sets (biased), BTFA (biased)	Comparator + / Comparator –<br>GPP +: 52 / 18 (verified 8+/0, 10 remain inconclusive)<br>GPP -: 3 (verified 2+/0, 1 remains inconclusive) / 1483
	Plesiomonas shigelloides	Culture	hugA PCR (biased), BTFA (biased)	Comparator + / Comparator –<br>GPP +: 3 / 15 (verified 15+/0)<br>GPP -: 0 / 1538
	Rotavirus	2x PCR	ECP (biased), BTFA (biased)	Comparator + / Comparator –<br>GPP +: 6 / 12 (verified 11+/0, 1 remains inconclusive)<br>GPP -: 0 / 1538

Study (GPP test)	Pathogen	Comparator	Verification method (judgement of bias)	Reported results of verification*
	Salmonella	Culture	stn PCR (biased)	Comparator + / Comparator –<br>GPP + 31 / 6 (verified 6+/0)<br>GPP - 0 / 1519
	Shigella	Culture	ECP (biased), BTFA (biased)	Comparator + / Comparator –<br>GPP + 47 / 2 (2 remain inconclusive)<br>GPP - 2 (2 remain inconclusive) / 1505
	STEC	2x PCR	ECP (biased), BTFA (biased)	Comparator + / Comparator –<br>GPP + 33 / 5 (verified 5+/0)<br>GPP - 0 / 1518
	Vibrio cholerae	Culture	gyrB PCR (biased), BTFA (biased)	Comparator + / Comparator –<br>GPP + 0 / 1 (verified 1+/0)<br>GPP - 0 / 1555
	Vibrio spp.	Culture	gyrB PCR (biased), BTFA (biased)	Comparator + / Comparator –<br>GPP + 0 / 2 (verified 2+/0)<br>GPP - 0 / 1554
	EAEC	2x PCR	ECP (biased), BTFA (biased)	Comparator + / Comparator –<br>GPP + 82 / 27 (verified 27+/0)<br>GPP - 1 (1 remains inconclusive) / 1446
	EPEC	2x PCR (of samples that were STEC negative)	ECP (biased), BTFA (biased)	Comparator + / Comparator –<br>GPP + 314 / 34 (verified 23+/0, 11 remain inconclusive)<br>GPP - 3 (3 remain inconclusive) / 1167
	Astrovirus	PCR	ECP (biased), BTFA (biased)	Comparator + / Comparator –<br>GPP + 7 / 1 (verified 1+/0)<br>GPP - 0 / 1548
	Sapovirus	2x PCR (one with published primers and one with in-house primers)	ECP (biased), BTFA (biased)	Comparator + / Comparator –<br>GPP + 46 / 13 (verified 12+/0, 1 remains inconclusive)<br>GPP - 0 / 1497
Deng 2015[10] (xTAG)	Adenovirus 40/41	Immunochromatography	Singleplex PCR and sequencing with primers specific for conserved regions of each target sourced from published literature (biased)	Comparator + / Comparator –<br>GPP + 3 / 0<br>GPP - 2 (verified 0/2-) / 285
	Campylobacter	Culture	Singleplex PCR and sequencing with primers specific for conserved regions of each target sourced	Comparator + / Comparator –<br>GPP + 20 / 16 (verified 16+/0)<br>GPP - 0 / 254

Study (GPP test)	Pathogen	Comparator	Verification method (judgement of bias)	Reported results of verification*		
			from published literature (biased)			
	E.coli O157	Culture confirmed by gene sequencing using published primers	Singleplex PCR and sequencing with primers specific for conserved regions of each target sourced from published literature (biased)		Comparator +	Comparator –
				GPP +	1	2 (verified 2+/0)
				GPP -	0	287
	ETEC	Culture confirmed by gene sequencing using published primers	Singleplex PCR and sequencing with primers specific for conserved regions of each target sourced from published literature (biased)		Comparator +	Comparator –
				GPP +	1	4 (verified 4+/0)
				GPP -	0	285
	Norovirus GII	Real-time reverse transcription PCR using PCR kit	Singleplex PCR and sequencing with primers specific for conserved regions of each target sourced from published literature (biased)		Comparator +	Comparator –
				GPP +	37	3 (verified 0/3-)
				GPP -	2 (verified 0/2+)	248
	Rotavirus	Immunochromatography	Singleplex PCR and sequencing with primers specific for conserved regions of each target sourced from published literature (unbiased)		Comparator +	Comparator –
				GPP +	61	6 (verified 6+/0)
				GPP -	1 (verified 0/1-)	222
	Salmonella	Culture confirmed by serotyping	Singleplex PCR and sequencing with primers specific for conserved regions of each target sourced from published literature (biased)		Comparator +	Comparator –
				GPP +	25	6 (verified 3+/3-)
				GPP -	5 (verified 5+/0)	254
	Shigella	Culture confirmed by serotyping	Singleplex PCR and sequencing with		Comparator +	Comparator –
				GPP +	3	1 (verified 1+/0)

Study (GPP test)	Pathogen	Comparator	Verification method (judgement of bias)	Reported results of verification*		
			primers specific for conserved regions of each target sourced from published literature (biased)	GPP -	0	286
FDA 2012[12] (xTAG)	Adenovirus	EIA and one PCR/ sequencing assay	Bi-directional sequencing analysis using analytically validated primers that targeted genomic regions distinct from the xTAG GPP (biased)	 GPP +<br>GPP -	Comparator +<br>4<br>1 (1+/0)	Comparator –<br>13<br>1154
	C. difficile toxin A/B	Bartels Cytotoxicity Assay for Clostridium difficile Toxin	Bi-directional sequencing analysis using analytically validated primers that targeted genomic regions distinct from the xTAG GPP (biased), **or** FDA cleared C. difficile Toxin molecular assays (biased)	 GPP +<br><br>GPP -	Comparator +<br>107<br><br>7	Comparator –<br>105 (verified 48+/0,<br>57 remain inconclusive)<br>922
	Campylobacter	Culture<br>(A PCR/Sequencing assay was also performed directly on clinical specimens that were tested positive by culture for species identification only)	Bi-directional sequencing analysis using analytically validated primers that targeted genomic regions distinct from the xTAG GPP (biased)	 GPP +<br><br>GPP -	Comparator +<br>3<br><br>0	Comparator –<br>21 (verified 6+/0,<br>15 remain inconclusive)<br>1155
	Cryptosporidium	Microscopy	Bi-directional sequencing analysis using analytically validated primers that targeted genomic regions distinct from the xTAG GPP (biased)	 GPP +<br><br>GPP -	Comparator +<br>12<br><br>1	Comparator –<br>53 (verified 8+/0,<br>45 remain inconclusive)<br>1131
	E.coli O157	Culture	Bi-directional sequencing analysis using analytically	 GPP +	Comparator +<br>2	Comparator –<br>9 (verified 4+/0,<br>5 remain inconclusive)

Study (GPP test)	Pathogen	Comparator	Verification method (judgement of bias)	Reported results of verification*		
			validated primers that targeted genomic regions distinct from the xTAG GPP (biased)	GPP -	0	1158
	ETEC	Composite consisting of PCR/sequencing directly from clinical specimen using four PCR/sequencing assays	Bi-directional sequencing analysis using analytically validated primers that targeted genomic regions distinct from the xTAG GPP (biased)		Comparator +	Comparator –
				GPP +	2 (verified 2+/0)	4
				GPP -	6 (verified 6+/0)	1156
	Norovirus	Composite consisting of CDC real-time PCR and conventional PCR followed by bi-directional sequencing assays directly from clinical specimen	Validated PCR/sequencing assays (biased)		Comparator +	Comparator –
				GPP +	74	96
				GPP -	4 (verified 0/4-)	1023
	Salmonella	Culture	Bi-directional sequencing analysis using analytically validated primers that targeted genomic regions distinct from the xTAG GPP (biased)		Comparator +	Comparator –
				GPP +	10	18 (verified 2+/0, 16 remain inconclusive)
				GPP -	0	1143
	Shigella	Culture	Bi-directional sequencing analysis using analytically validated primers that targeted genomic regions distinct from the xTAG GPP (biased)		Comparator +	Comparator –
				GPP +	2	17 (verified 2+/0, 15 remain inconclusive)
				GPP -	0	1154
	STEC	Broth enrichment followed by rapid immunoassay	Bi-directional sequencing analysis using analytically validated primers that targeted		Comparator +	Comparator –
				GPP +	1	16 (verified 1+/0, 15 remain inconclusive)
				GPP -	0	1153

Study (GPP test)	Pathogen	Comparator	Verification method (judgement of bias)	Reported results of verification*
			genomic regions distinct from the xTAG GPP (biased)	
Pankhurst 2014[16] (xTAG)	Campylobacter	Culture	single qPCR: primers probably different to xTAG (biased)	Comparator + / Comparator –<br>GPP + 110 / 11 (verified 11+/0)<br>GPP - 7 (verified 3+/0, 4 remain inconclusive) / 711
	C. difficile toxin A and B	EIA plus culture	single qPCR: primers probably different to xTAG (biased)	Comparator + / Comparator –<br>GPP + 195 / 19 (verified 15+/0, 4 remain inconclusive)<br>GPP - 4 (verified 2+/0, 2 remain inconclusive) / 621
	Norovirus	Quantitative PCR	single qPCR (biased)	Comparator + / Comparator –<br>GPP + 183 / 16 (verified 7+/0, 9 remain inconclusive)<br>GPP - 16 (verified 6+/0, 10 remain inconclusive) / 624
	Salmonella	Culture	single qPCR: primers probably different to xTAG (biased)	Comparator + / Comparator –<br>GPP + 15 / 9 (verified 0+/0, 9 remain inconclusive)<br>GPP - 18 (verified 10+/0, 8 remain inconclusive) / 797

ECP enhanced comparator protocol (original molecular comparator assays with enhanced methods, including up to 10 additional PCR cycles and up to 10 additional replicate samples); BTFA benchtop version of the FilmArray GI Panel assay (FilmArray primers in a conventional real-time PCR) followed by amplicon sequencing; GPP gastrointestinal pathogen panel; qPCR quantitative polymerase chain reaction; EIA enzyme immunoassay; *C. difficile Clostridium difficile*; ETEC Enterotoxigenic *Escherichia coli*; E.coli O157 *Escherichia coli* O157; STEC Shiga toxin-producing *Escherichia coli*; EAEC Enteroaggregative *Escherichia coli*; EPEC Enteropathogenic *Escherichia coli*; (green – verification in favour of GPP, red – verification in favour of comparator)

*Reported results of verification is presented as 2x2 comparison table for GPP and routine testing. Interpretation is as follows using Buss 2015, adenovirus as an example: 42 positive results and 1499 negative results were in agreement using both methods. Thirteen discordant GPP positive/ comparator negative tests were verified using ECP and BFTA, 11 were found to be positive and 2 inconclusive using this third method. Two discordant GPP negative / comparator positive were verified using ECP and BFTA, 1 was found to be positive and 1 was inconclusive using this third method.

** Inconclusive – discrepant analysis did not help identify the underlying cause of the discrepancy
